# Supplementary material for: The solute carrier superfamily interactome
Source: Mol Syst Biol. 2025 May 12;21(6):632–75. doi: 10.1038/s44320-025-00109-1 (PMC12130317; doi:10.1038/s44320-025-00109-1)
Supplement: Supplementary file 1 — Appendix [file 44320_2025_109_MOESM1_ESM.docx]

**Appendix**

The solute carrier superfamily interactome

*Fabian Frommelt^1,#^, Rene Ladurner^1,#^, Ulrich Goldmann^1^, Gernot Wolf^1^, Alvaro Ingles-Prieto^1^, Eva Lineiro-Retes^1^, Zuzana Gelová^1^, Ann-Katrin Hopp^1^, Eirini Christodoulaki^1^, Shao Thing Teoh^1^,* *Philipp Leippe^1^, Brianda L. Santini^1^, Manuele Rebsamen^1^, Sabrina Lindinger^1^, Iciar Serrano^1^, Svenja Onstein^1^, Christoph Klimek^1^, Barbara Barbosa^1^, Anastasiia Pantielieieva^1^, Vojtech Dvorak^1^, J. Thomas Hannich^1^, Julian Schoenbett^2^, Gilles Sansig^2^, Tamara A.M. Mocking^3^, Jasper F. Ooms^3^, Adriaan P. IJzerman^3^, Laura H. Heitman^3^, Peter Sykacek^4^, Juergen Reinhardt^2^, André C Müller^1^, Tabea Wiedmer^1^, Giulio Superti-Furga^1,5,6*^*

**Affiliations:**

^1^ CeMM Research Center for Molecular Medicine of the Austrian Academy of Sciences, 1090 Vienna, Austria

^2^ Novartis Pharma AG, Novartis Biomedical Research NBR/DSc, CH-4002 Basel, Switzerland

^3^ Division of Drug Discovery and Safety, Leiden Academic Centre for Drug Research, Leiden University, Einsteinweg 55, 2333 CC Leiden, The Netherlands

^4^ Department of Biotechnology, University of Natural Resources and Life Sciences, 1190 Vienna, Austria

^5^ Center for Physiology and Pharmacology, Medical University of Vienna, 1090 Vienna, Austria

^6^ Fondazione Ri.MED, Palermo, Italy

^#^ Equal contribution

^*^ Corresponding author

Corresponding author: Giulio Superti-Furga, CeMM Research Center for Molecular Medicine of the Austrian Academy of Sciences, Lazarettgasse 14, AKH BT 25.3, 1090 Vienna, Austria; email:  gsuperti@cemm.oeaw.ac.at, phone: +43 1 40160 70001

**Table of Content**

[Appendix Figures 3](#_Toc192510301)

[Appendix Figure S1. General properties of SLC baits and total signal in the SLC-interactome. 3](#_Toc192510302)

[Appendix Figure S2. Distribution of preys per SLC AP-MS experiment before and after scoring and quantitative characteristics of the scored SLC-interactome. 4](#_Toc192510303)

[Appendix Figure S3. Prediction of the PPI-structure of SLC16-family chaperone interactions SLC16A7-BSG and SLC16A1-EMB by AlphaFold multimer. 5](#_Toc192510304)

[Appendix Figure S4. Structural prediction of SLC-chaperone interactions of SLC7A7-SLC3A2. 6](#_Toc192510305)

[Appendix Figure S5. Structural prediction of SLC-chaperone interactions of SLC9A2-CHP1. 7](#_Toc192510306)

[Appendix Figure S6. Prediction of the structure of SLC30A1 interactions with GALNT2 by AlphaFold multimer. 8](#_Toc192510307)

[Appendix Figure S7. Assessment of quality of hierarchical clustering of SLC-interactome profiles by the mean silhouette width. 8](#_Toc192510308)

[Appendix Figure S8. Functional enrichment analysis of clusters obtained from the SLC-interactome profile analysis. 9](#_Toc192510309)

[Appendix Figure S9. Co-purification analysis in the SLC-interactome to deconvolute interactome organization to protein complexes. 10](#_Toc192510310)

[Appendix Figure S10. Protein Stability after RNAi-mediated depletion of interactor and additional drug treatment. 12](#_Toc192510311)

[Appendix Figure S11. Protein Stability after cDNA-mediated overexpression of interactor and additional drug treatment. 14](#_Toc192510312)

[Appendix Figure S12. SLC16A6 binds to an SCF E3 ubiquitin-protein ligase complex which mediates abundance. 15](#_Toc192510313)

[Appendix Figure S13. Correlation of relative fluorescence changes after depletion of SLC interactors in protein stability and subcellular localization assays (Fig. 4, Fig. 6). 17](#_Toc192510314)

[Appendix Figure S14. Transporter function of SLC1A3 and SLC22A3 is affected by the depletion of distinct interactors. 18](#_Toc192510315)

[Appendix Figure S15. Transporter assay results for SLC39A8 after RNAi of selected interaction partners. 19](#_Toc192510316)

[Appendix Figure S16. Prediction of the structure of SLC43A2 interactions with LIN7C and MPP1 by AlphaFold multimer 20](#_Toc192510317)

[Appendix Figure S17. Coverage of endogenously expressed SLCs in transcriptome, full proteome profiling of HEK 293 Jump-In compared to SLC-interactome. 21](#_Toc192510318)

[Appendix Figure S18. Validation of 9 DsiRNA pools used for validation of SLC-protein interactions. 22](#_Toc192510319)

[Appendix Figure S19. Assessment of RNAi efficiency targeting three SLC interaction partners by full proteome profiling in HEK 293 Jump In T-REx cells. 23](#_Toc192510320)

[References 24](#_Toc192510321)

# Appendix Figures


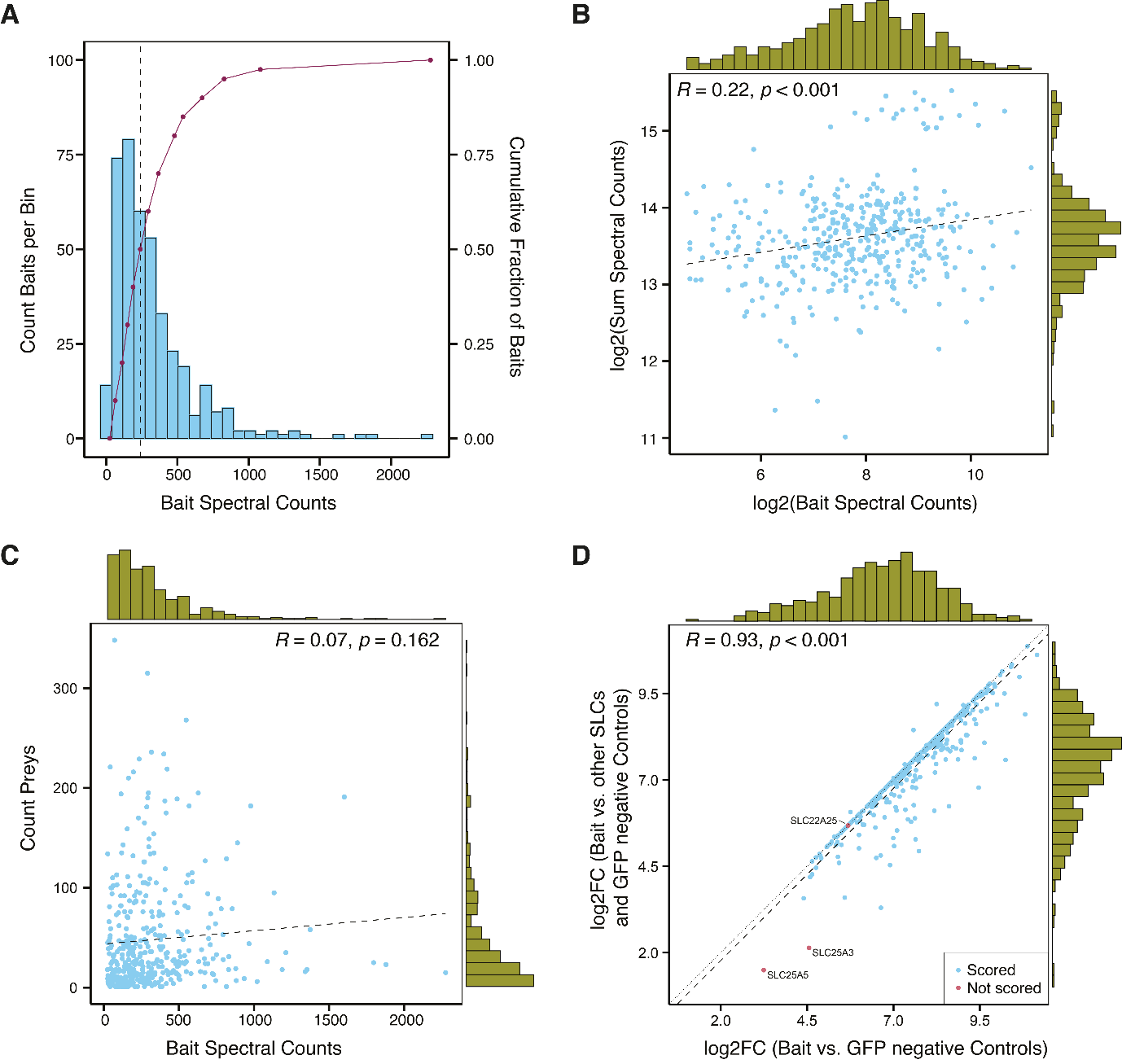


Appendix Figure S1. General properties of SLC baits and total signal in the SLC-interactome. **(A)** Distribution of SLC bait spectral counts (blue) for each AP-MS in the SLC-interactome. The median bait signal was 239 spectral counts (black dashed line), with a 25% quantile of 129.5 and a 75% quantile of 406 spectral counts. The right y-axis indicates cumulative fraction of baits in percentage and the purple line shows the fraction of baits across the SLC-interactome dataset. **(B)** log2 of bait spectral count versus log2 of the summed spectral count per sample (n=405). Marginal histograms of data distribution are indicated. Pearson correlation coefficient, R=0.22, p<0.001 shows no correlation. **(C)** Average spectral count signal per bait plotted against the count of interactions scored per bait protein. Marginal histograms of data distribution are indicated (n=405). Pearson correlation coefficient, R=0.07, p=0.162 shows no correlation. **(D)** For each bait protein the log2FC versus GFP negative controls (x-axis) is plotted against the log2FC versus the average spectral counts of the bait protein quantified within the rest of the interactome samples (SLCs and GFP negative controls). Some baits show a lower enrichment against the other SLC AP-MS samples compared to the enrichment against GFP negative controls, indicating that the log2FC derived against the other SLC AP-MS samples is a more conservative measure to filter background of SLC AP-MS experiments, thus modulating stricter the SLC/TMprotein- specific background. Three SLCs (SLC25A3, SLC25A5, SLC22A25) did not pass the scoring probability threshold (red dots), whereas 402 baits were found significantly scored (blue dots). The black dotted line indicates a linear regression passing through the origin, and the black dotted line indicates the linear regression and the blacked line the Pearson correlation (Peason correlation coefficient, R=0.93, p-value < 0.01). The marginal distributions are indicated as histograms.


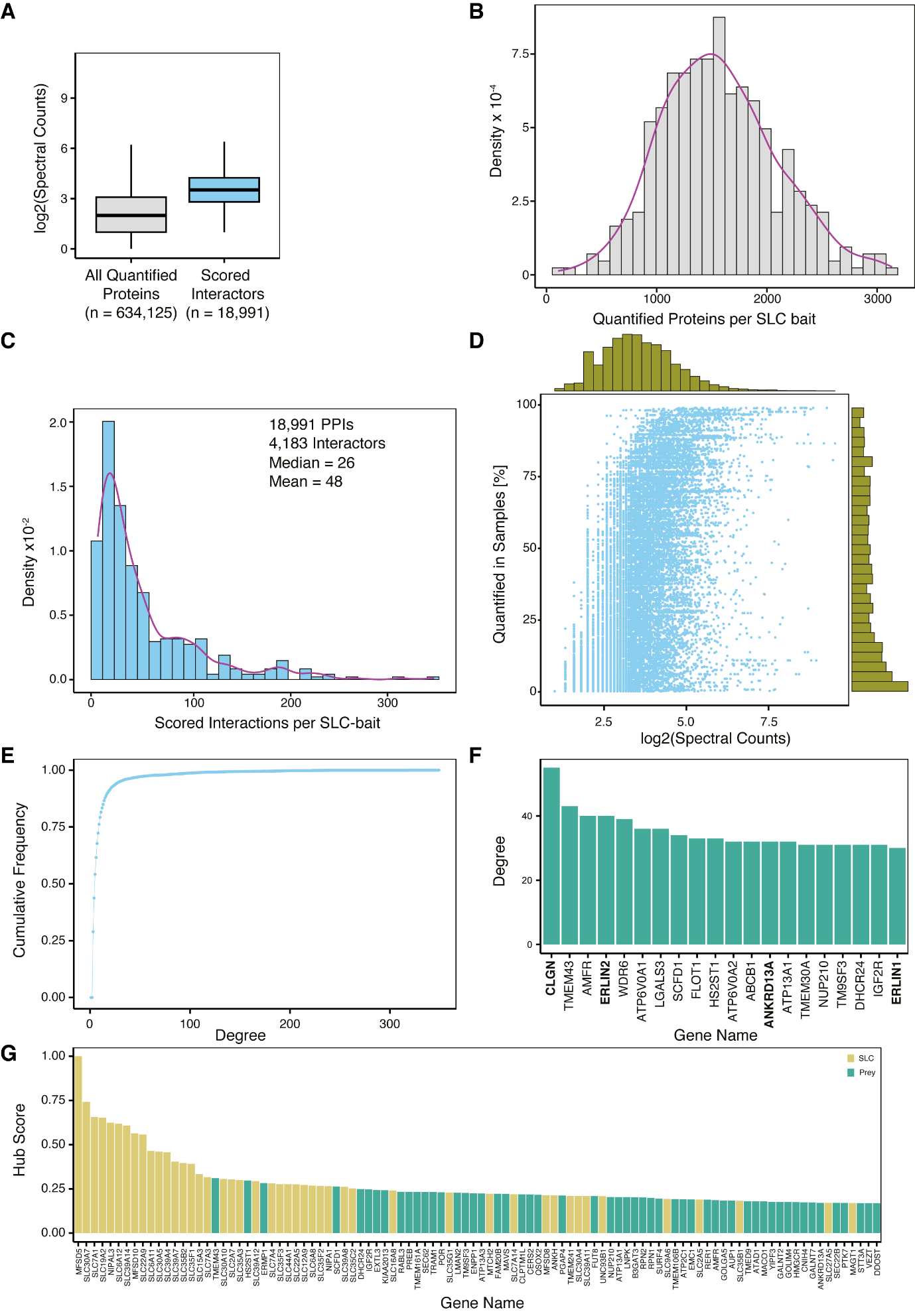


Appendix Figure S2. Distribution of preys per SLC AP-MS experiment before and after scoring and quantitative characteristics of the scored SLC-interactome. **(A)** Boxplot of all proteins which are not scored (n=634,125, grey) and of scored interactors (n=18,991, blue). Lower and upper hinges of box plots correspond to the 25^th^ and 75^th^ percentiles, respectively. Lower and upper whiskers extend from the hinge to the smallest or largest value no further than the 1.5× interquartile range from the hinge, respectively. Black line represents the median. For better readability outliers were removed. Spectral counts (y-axis) were log2 transformed. **(B)** Distribution of proteins per SLC-protein before filtering. **(C)** Distribution of scored interaction partners per SLC. **(D)** Scored protein interactions plotted against the recovery across the SLC-interactome. The log2 of the average spectral counts (x-axis) are plotted against the percentage of quantification across the SLC-interactome. Marginal histograms of data distribution are indicated (n=18,991)**. (E)** Cumulative degree (y-axis) of all proteins within the PPI-network of the SLC interactome. **(F)** Top 20 most connected interaction partners sorted by the degree of each network node (y-axis). The interactors associated with proteostatic regulation of SLCs are marked in bold. **(G)** Representation of the 100 most connected hubs in the SLC-interactome ranked by the highest hub-score (y-axis). SLCs are colored yellow and interactors in green.


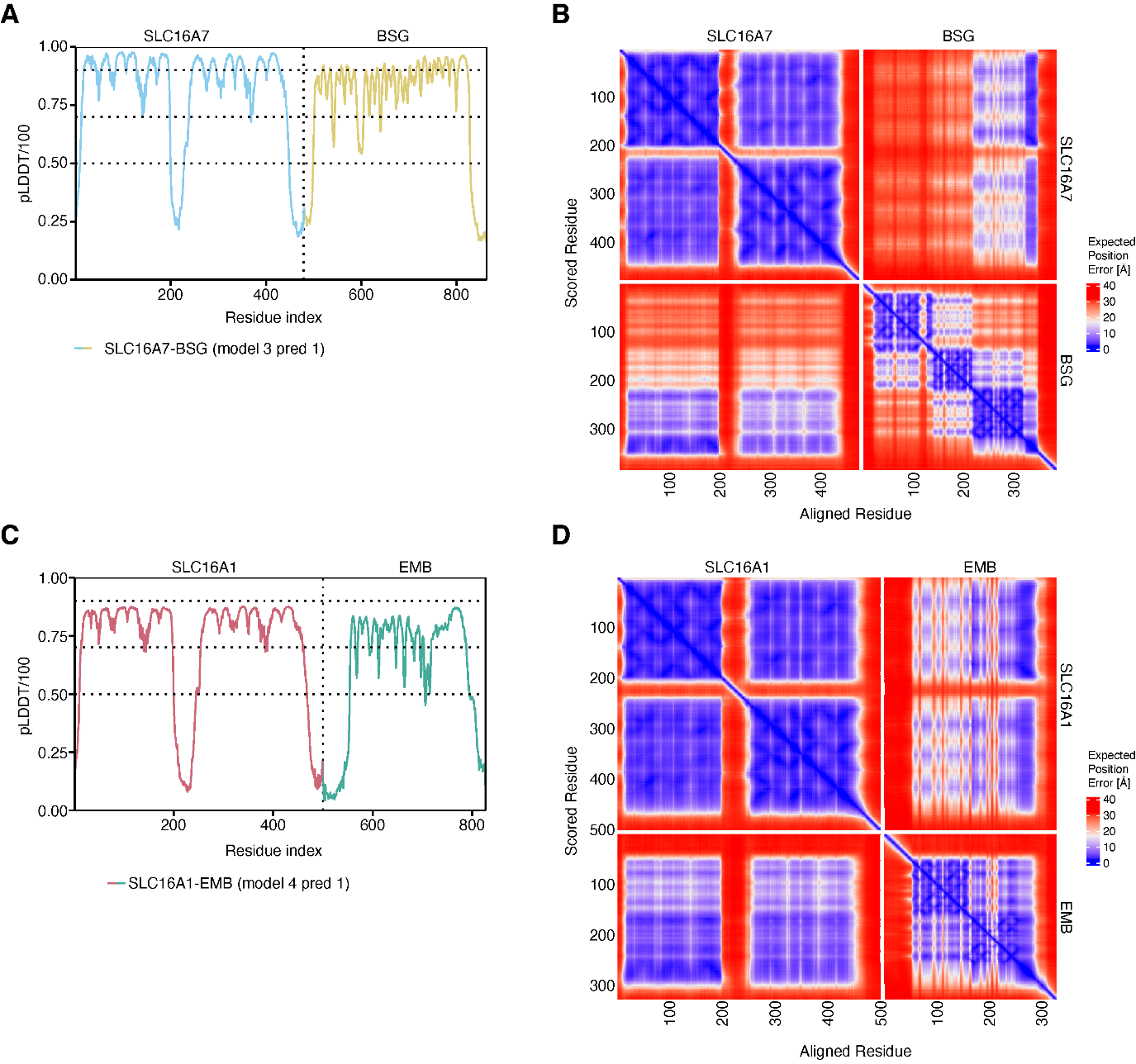


Appendix Figure S3. Prediction of the PPI-structure of SLC16-family chaperone interactions SLC16A7-BSG and SLC16A1-EMB by AlphaFold multimer. **(A)** AlphaFold multimer confidence in pLDDT/100 for best ranked model of the SLC16A7-BSG interaction. **(B)** Inter PAE (Predicted Aligned Error) heatmap for the best ranked model of the SLC16A7-BSG interaction. **(C)** AlphaFold multimer confidence in pLDDT/100 for best ranked model of the SLC16A1-BSG interaction. **(D)** Inter PAE heatmap for the best ranked structural prediction of the SLC16A1-EMB interaction.


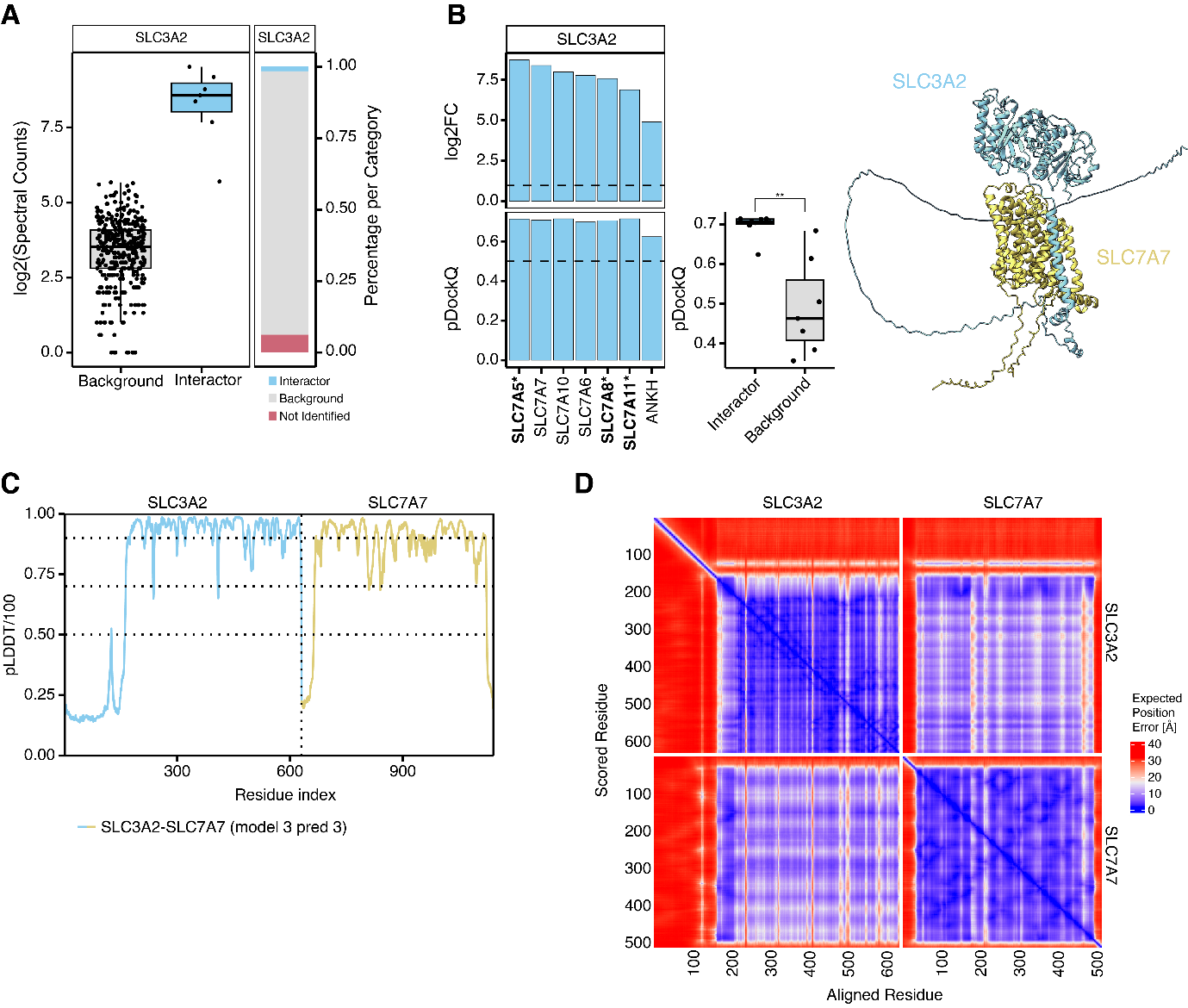


Appendix Figure S4. Structural prediction of SLC-chaperone interactions of SLC7A7-SLC3A2. **(A)** Distribution of SLC3A2 across the SLC-interactome. The left panel shows the log2 transformed SPC for each SLC AP-MS experiment separated by scored interactions (n=7, blue) and background/ not interacting (n=372, grey). On the right side it is indicated how often SLC3A2 was identified, scored or found as background. Lower and upper hinges of box plots correspond to the 25^th^ and 75^th^ percentiles, respectively. Lower and upper whiskers extend from the hinge to the smallest or largest value no further than the 1.5× interquartile range from the hinge, respectively. Black line represents the median log2 spectral count signal, and the black dots represent the signal per measurement. **(B)** The upper bar chart shows all SLCs for which SLC3A2 was scored within the SLC-interactome (log2FC against GFP, threshold of log2FC > 1). The lower section of the bar chart shows the confidence scores of predicted SLC3A2-SLC complexes (high confidence, pDockQ threshold of > 0.5 indicated by dashed line). Complexes for which the experimental structure was solved are marked with an asterisk (“*”). Predicted complex structures of interactions (n=7) were compared against a negative set of SLC-chaperone complexes (n=7; unpaired student t-test, p-value=0.0009177). In the figure panel, p-values below 0.01 are indicated with “**”. Lower and upper hinges of box plots correspond to the 25^th^ and 75^th^ percentiles, respectively. Lower and upper whiskers extend from the hinge to the smallest or largest value no further than the 1.5× interquartile range from the hinge, respectively. Black line represents the median and the black dots represent scores per complex. On the right side the model of SLC7A7-SLC3A2 is shown. **(C)** AlphaFold multimer confidence in pLDDT/100 for best ranked model of the hetromer SLC7A7-SLC3A2. **(D)** Inter PAE (Predicted Aligned Error) heatmap for the best ranked model of SLC7A7-SLC3A2.


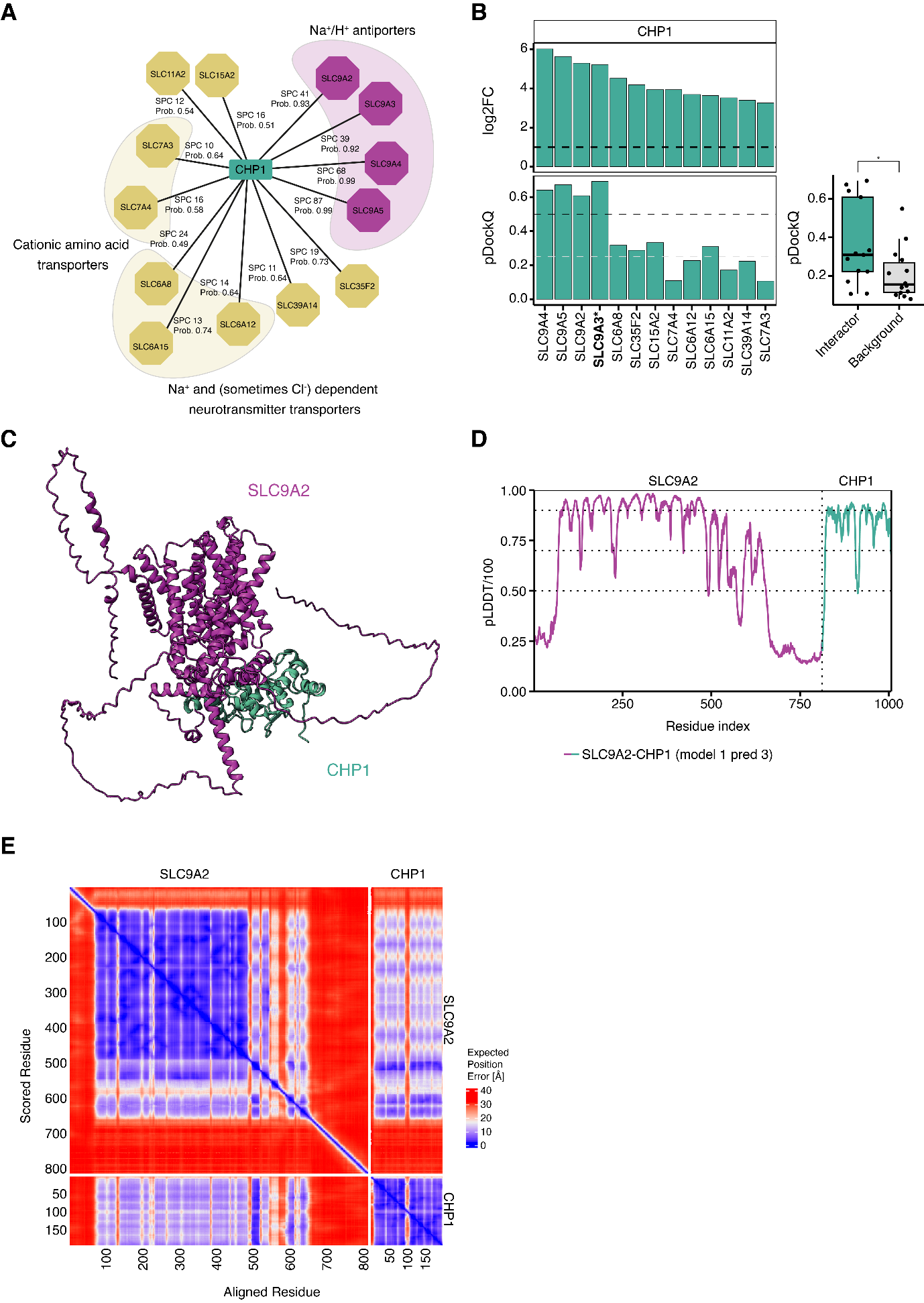


Appendix Figure S5. Structural prediction of SLC-chaperone interactions of SLC9A2-CHP1. **(A)** SLC-CHP1 interactions in the SLC-interactome (teal). Na+/H+ antiporters of the SLC9-family are highlighted in purple, other SLCs are grouped by family and coloured in yellow. **(B)** Log2FC against GFP and pDockQ-score of structural models for each SLC-CHP1 complex. Experimentally solved structures are marked in bold and with an asterisk (*). For CHP1 interactions with SLC9-family members (purple) high confidence models (dashed black line pDockQ > 0.5) were obtained, whereas for the other CHP1-SLC interaction (yellow) only medium (dashed grey line pDockQ > 0.25) to low confidence structures were found. A comparison against randomly sampled SLC-CHP1 interaction showed, a significant difference between models of the interactions covered in the SLC-interactome and the control set (unpaired student t-test, p-value=0.03642). Lower and upper hinges of box plots correspond to the 25^th^ and 75^th^ percentiles, respectively. Lower and upper whiskers extend from the hinge to the smallest or largest value no further than the 1.5× interquartile range from the hinge, respectively. Black line represents the median and the black dots represent scores per complex. **(C)** The model of SLC9A2-CHP1 is shown on the right side. **(D)** AlphaFold multimer confidence in pLDDT/100 for best ranked model of SLC9A2 interacting with the chaperone CHP1. **(E)** Inter PAE heatmap for the best ranked structural prediction of SLC9A2-CHP1 interaction.


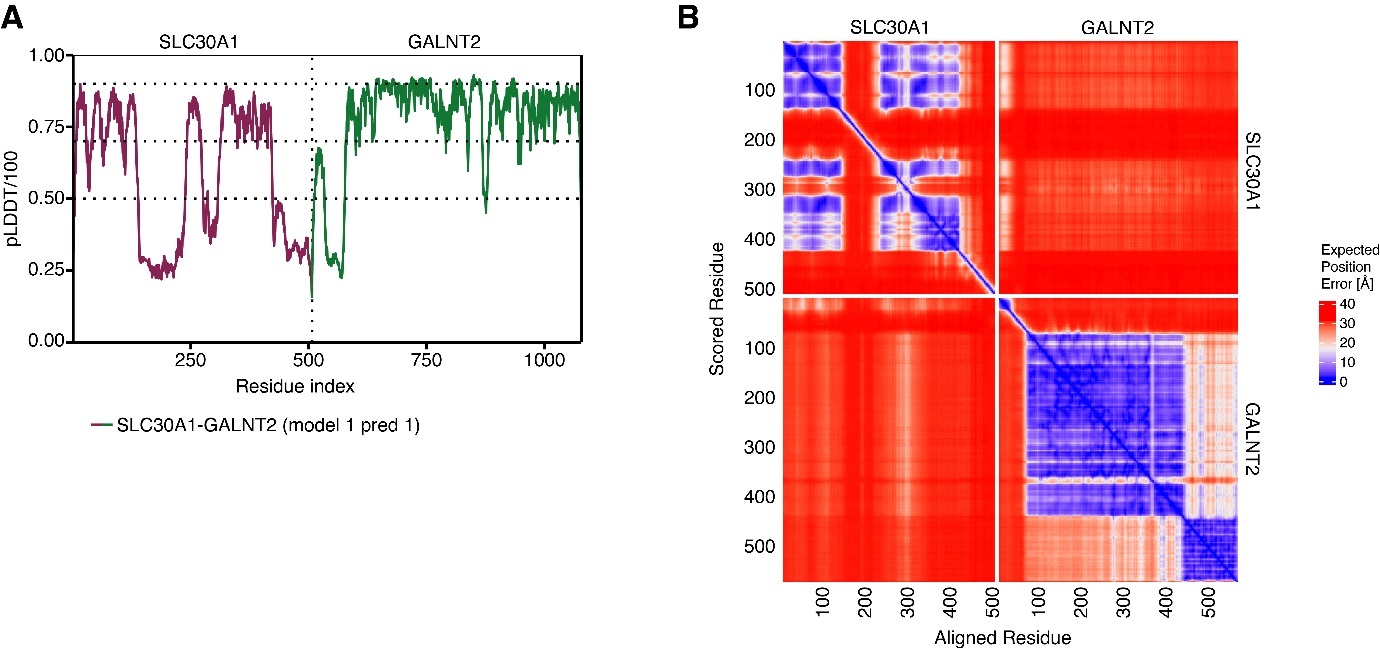


Appendix Figure S6. Prediction of the structure of SLC30A1 interactions with GALNT2 by AlphaFold multimer. **(A)** AlphaFold multimer confidence in pLDDT/100 for best ranked model of the SLC30A1-GALNT2 interaction. **(B)** Inter PAE heatmap for the best ranked model of the SLC30A1-GALNT2 interaction.


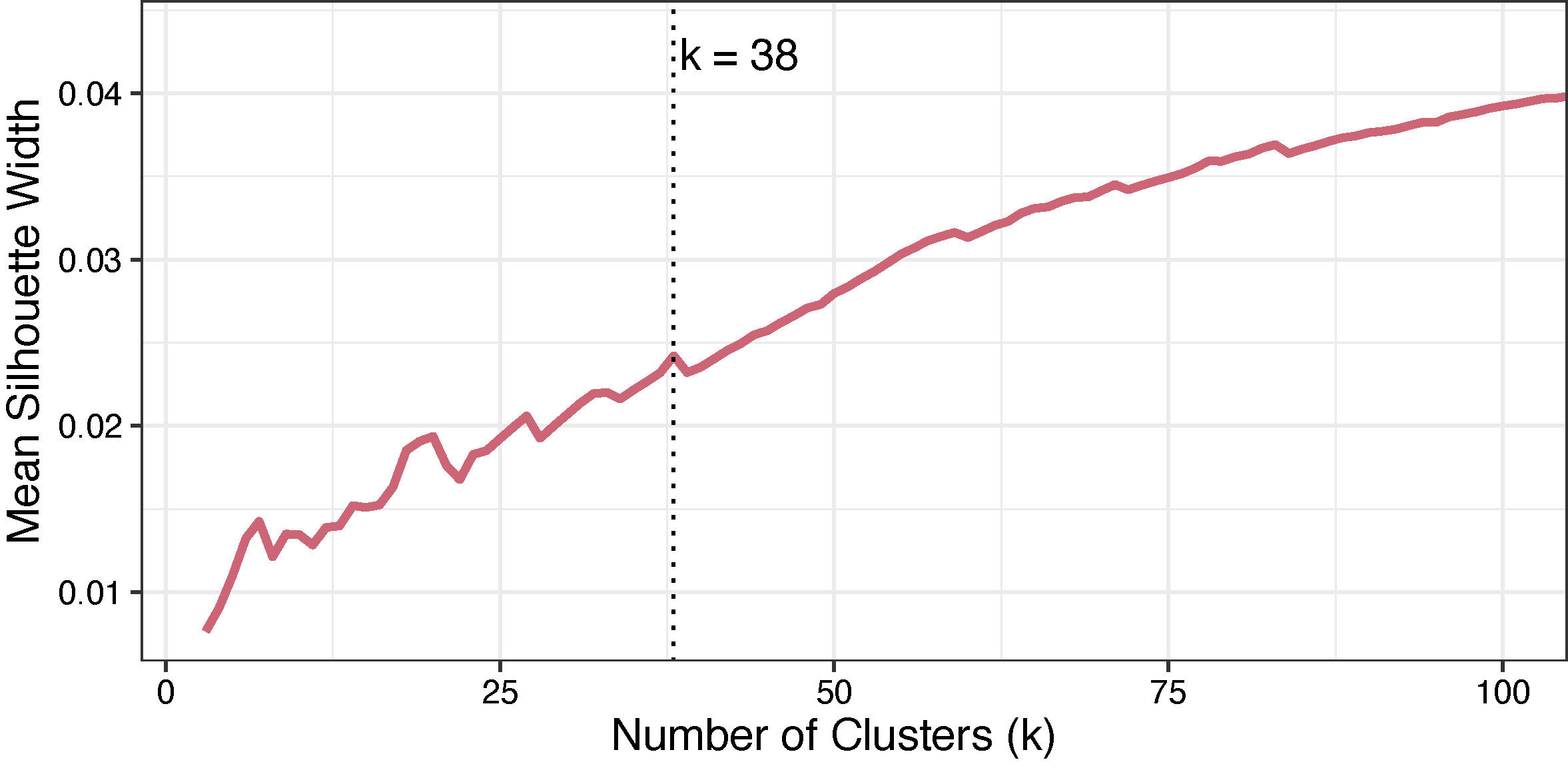


Appendix Figure S7. Assessment of quality of hierarchical clustering of SLC-interactome profiles by the mean silhouette width. The mean silhouette width per cluster with a k=3 to k=100 was calculated, and local maxima were identified. The local maxima at k=38 (black dotted line) was selected.


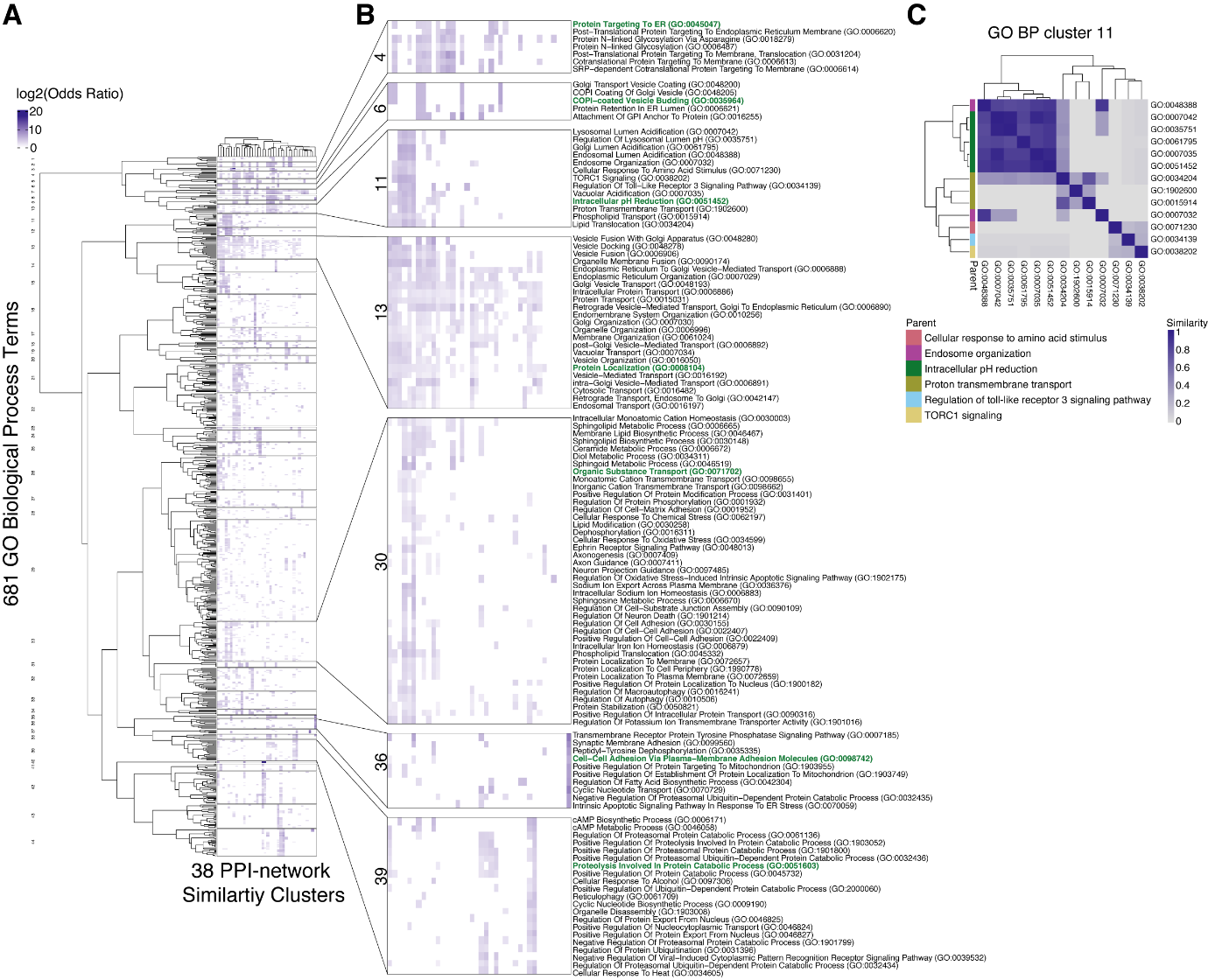


Appendix Figure S8. Functional enrichment analysis of clusters obtained from the SLC-interactome profile analysis. **(A)** Heatmap of significantly enriched GO biological processing terms (p-value < 0.01) across the 38 SLC-interactome profile similarity analysis. The heatmap was grouped by hierarchical clustering (Ward d2) into 44 clusters using Euclidian distance. An uncropped heatmap is provided in the source data. **(B)** Enlarged clusters from the heatmap are used to highlight term clusters used for subsequent determination of parental GO term for **Fig. 3A**. Parental terms are marked in dark green and bold. **(C)** TOP: GO BP cluster 11 similarity matrix. Bottom: the most frequent parental GO terms represented by cluster 11.


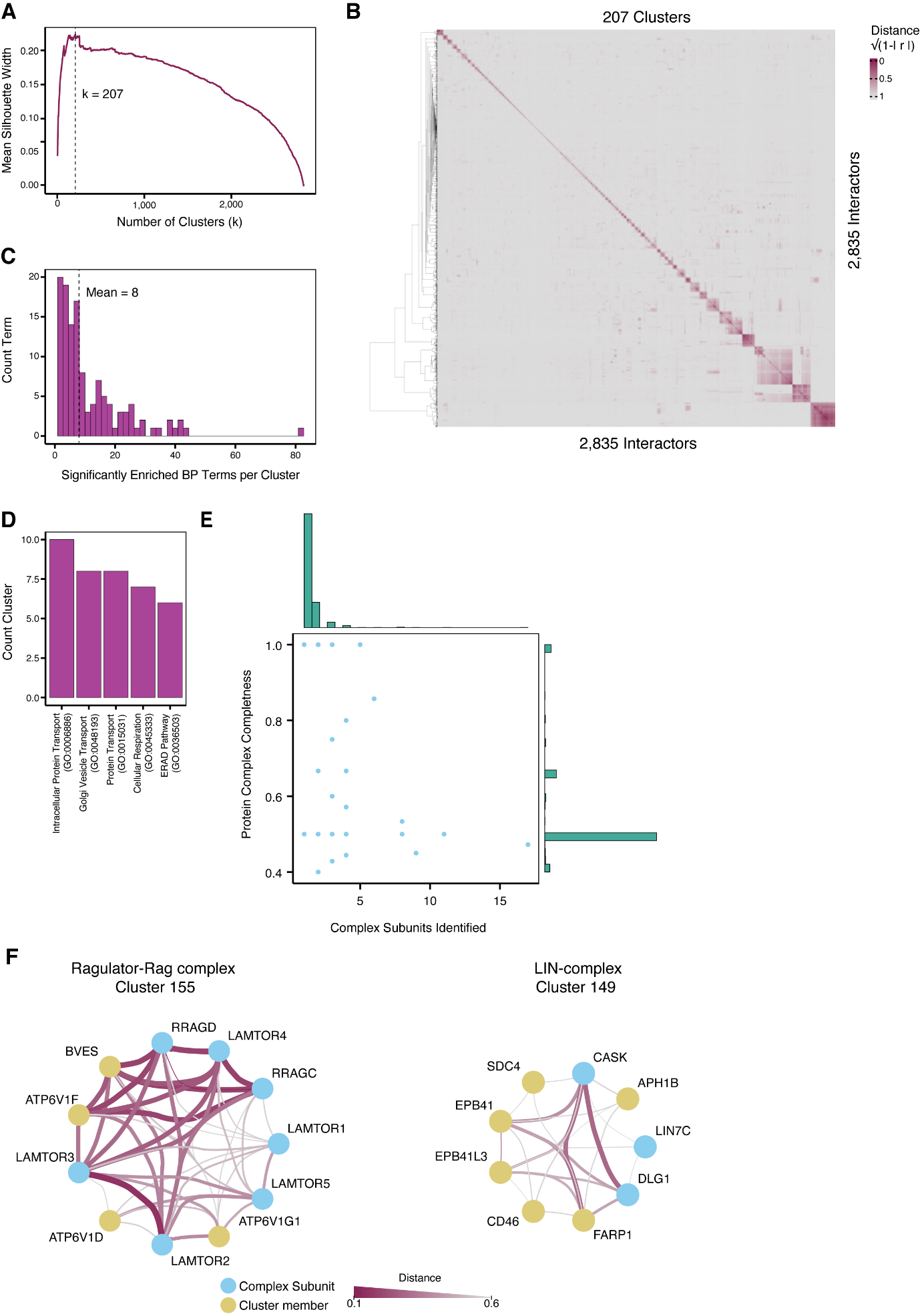


Appendix Figure S9. Co-purification analysis in the SLC-interactome to deconvolute interactome organization to protein complexes. **(A)** The mean silhouette width per cluster with a k=2 to k= 2834 was calculated, and local maxima were identified. The local maxima at k=207 (black dotted line) was selected. **(B)** Distance based prey-prey correlation heatmap. Clusters represent proteins, which are co-purified within multiple SLC AP-MS experiments. **(C)** Distribution of significantly enriched terms per cluster. On average, eight terms were significantly enriched per cluster (showed as dotted line). **(D)** Top 5 significantly scored BP terms across clusters. **(E)** Overview of copurified protein complexes. The number of identified subunits is plotted against the protein complex completeness. Marginal histograms of data distribution are indicated. **(F)** Ragulator-Rag complex (Complex IDs: 8325, 8324, 8324, 8306) and LIN-complex (Complex IDs: 617, 1448, 3207, 3208, 3209, 6263) based on distance matrix derived from prey-prey correlation. Line thickness represents density between as a measure of correlation as used in panel B. Color correlates with density values. Complex subunits are colored blue, while other proteins present in the cluster are colored yellow.


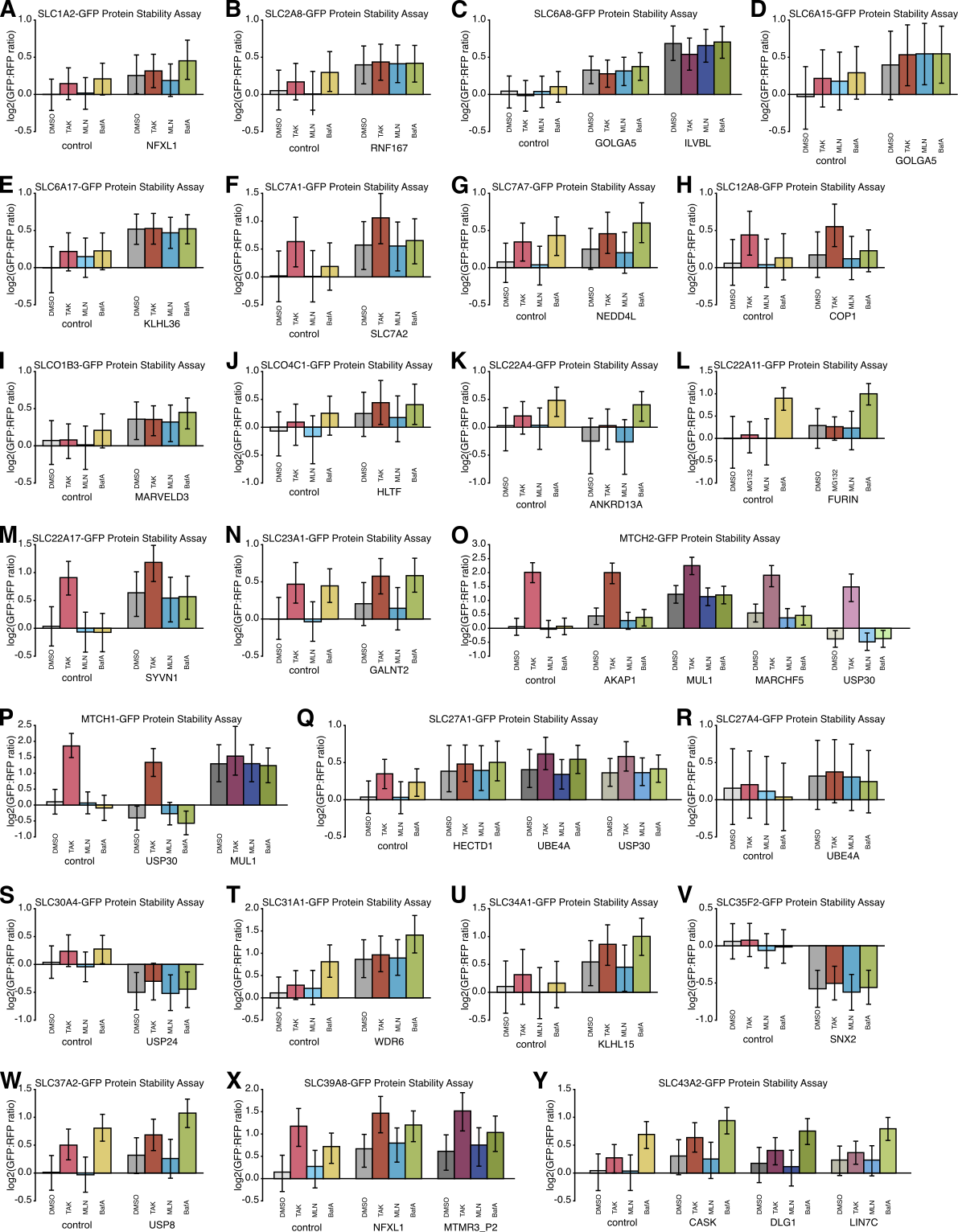


Appendix Figure S10. Protein Stability after RNAi-mediated depletion of interactor and additional drug treatment. All experiments were performed alongside the protein stability experiments shown in Figure 4D with an additional drug treatment of 4.5–6 hours before analysis (DMSO, 0.05% DMSO; TAK, 0.5 **µ**M TAK-243; MLN, 0.5 **µ**M MLN4924; BafA, 100 nM Bafilomycin A1; MG132, 10 **µ**M MG132). Graphs show median GFP:RFP ratio compared to control RNAi without drugs. Error bars denote upper and lower quartile. **(A)** SLC1A2-GFP protein stability assay (n>9670 events per sample). **(B)** SLC2A8-GFP protein stability assay (n>9899) events per sample). **(C)** SLC6A8-GFP protein stability assay (n>4588 events per sample). **(D)** SLC6A15-GFP protein stability assay (n>8513 events per sample). **(E)** SLC6A17-GFP protein stability assay (n>9496 events per sample). **(F)** SLC7A1-GFP protein stability assay (n>9210 events per sample). **(G)** SLC7A7-GFP protein stability assay (n>9848 events per sample). **(H)** SLC12A8-GFP protein stability assay (n>9321 events per sample). **(I)** SLCO1B3-GFP protein stability assay (n>8129 events per sample). **(J)** SLCO4C1-GFP protein stability assay (n>5538 events per sample). **(K)** SLC22A4-GFP protein stability assay (n>8309 events per sample). **(L)** SLC22A11-GFP protein stability assay (n>10010 events per sample). Note that proteasome inhibitor MG132 was used in this assay instead of TAK-243. **(M)** SLC22A17-GFP protein stability assay (n>9527 events per sample). **(N)** SLC23A1-GFP protein stability assay (n>5969 events per sample). **(O)** MTCH2-GFP protein stability assay (n>6005 events per sample). **(P)** MTCH1-GFP protein stability assay (n>4376 events per sample). **(Q)** SLC27A1-GFP protein stability assay (n>3813 events per sample). **(R)** SLC27A4-GFP protein stability assay (n>9963 events per sample). **(S)** SLC30A4-GFP protein stability assay (n>2342 events per sample). **(T)** SLC31A1-GFP protein stability assay (n>6839 events per sample). **(U)** SLC34A1-GFP protein stability assay (n>8777 events per sample). **(V)** SLC35F2-GFP protein stability assay (n>8798 events per sample). **(W)** SLC37A2-GFP protein stability assay (n>9620 events per sample). **(X)** SLC39A8-GFP protein stability assay (n>6809 events per sample). **(Y)** SLC43A2-GFP protein stability assay (n>9674 events per sample).


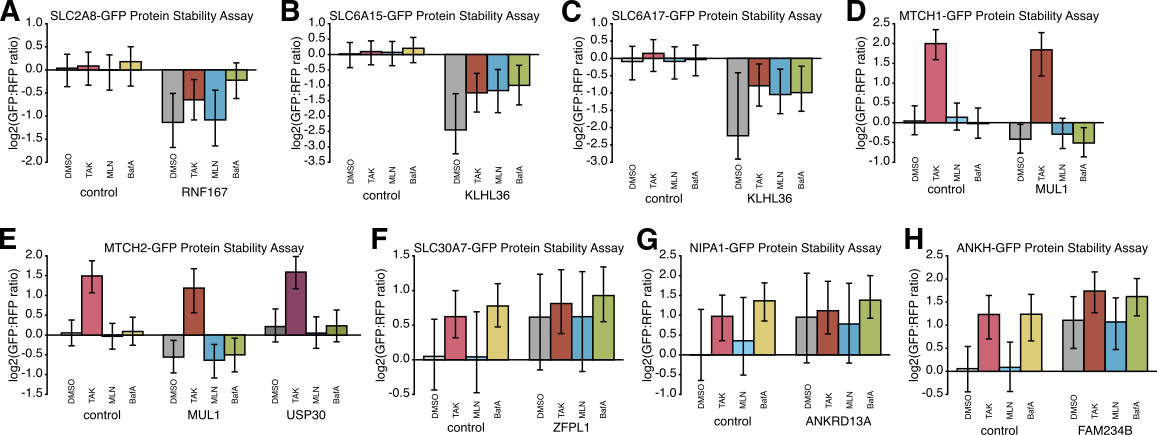


Appendix Figure S11. Protein Stability after cDNA-mediated overexpression of interactor and additional drug treatment. All experiments were performed alongside the protein stability experiments shown in **Fig. 4E** with an additional drug treatment of 4.5–6 hours before analysis (DMSO, 0.05% DMSO; TAK, 0.5 µM TAK-243; MLN, 0.5 µM MLN4924; BafA, 100 nM Bafilomycin A1). Graphs show median GFP:RFP ratio compared to BFP overexpression without drugs. Error bars denote upper and lower quartile. **(A)** SLC2A8-GFP protein stability assay (n>4606 events per sample). **(B)** SLC6A15-GFP protein stability assay (n>3297 events per sample). **(C)** SLC6A17-GFP protein stability assay (n>1615 events per sample). **(D)** MTCH1-GFP protein stability assay (n>3006 events per sample). **(E)** MTCH2-GFP protein stability assay (n>1673 events per sample). **(F)** SLC30A7-GFP protein stability assay (n>4680 events per sample). **(G)** NIPA1-GFP protein stability assay (n>2872 events per sample). **(H)** ANKH-GFP protein stability assay (n>3490 events per sample).


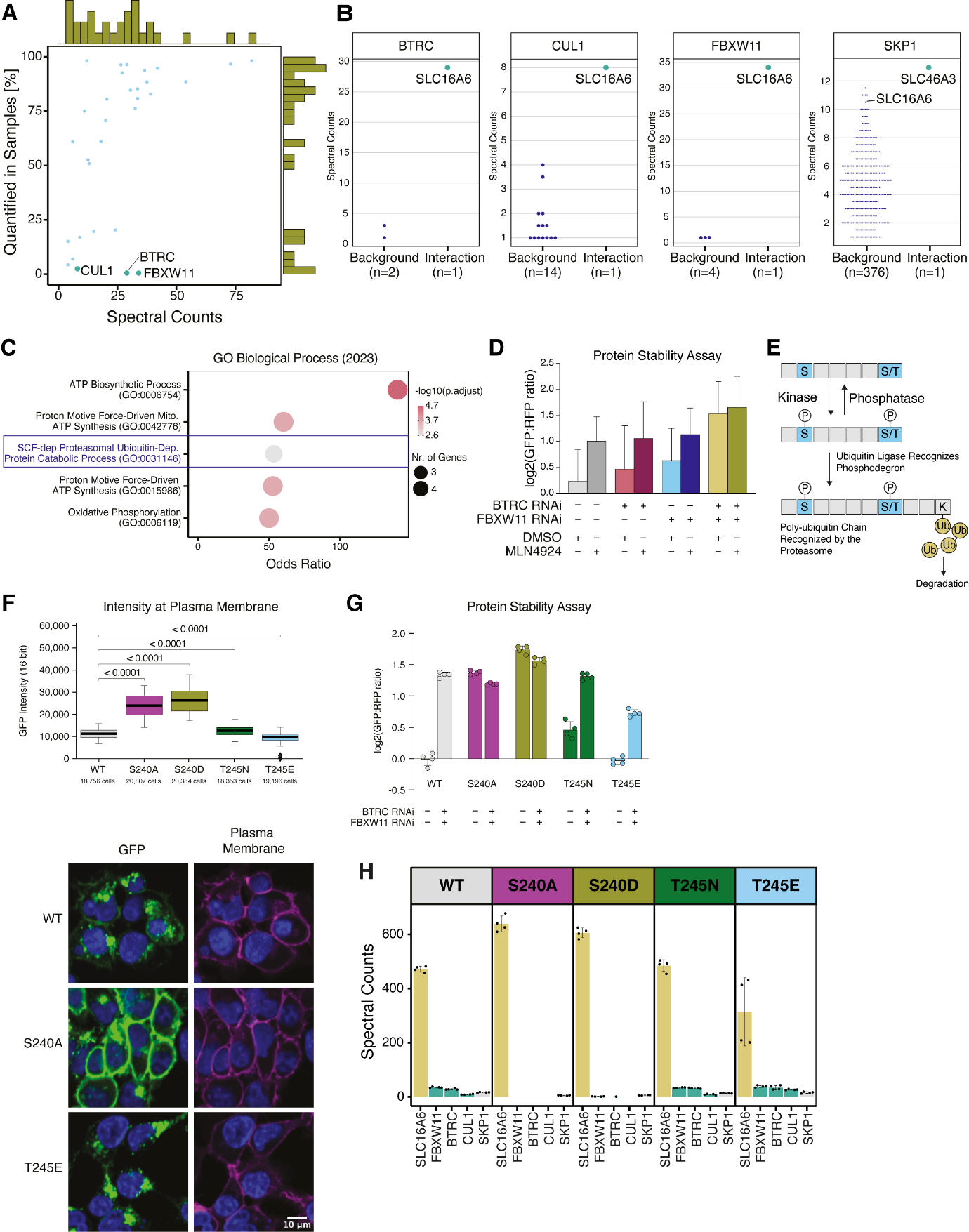


Appendix Figure S12. SLC16A6 binds to an SCF E3 ubiquitin-protein ligase complex which mediates abundance. **(A)** Scored interactors (blue points, n=31) of SLC16A6 plotted against identification across the SLC-interactome in percentage. Marginal histograms of data distribution are indicated on the x- and y-axis. SCF complex subunits are labelled. **(B)** Abundance distribution and interactor scoring results of SCF complex members BTRC, CUL1, FBXW11 and SKP1. SKP1 was found in the background of the SLC16A6 purification. **(C)** Five most significant GO biological processing terms enriched in the interactome of SLC16A6. **(D)** Protein stability assay of SLC16A6^WT^ after depletion of BTRC and FBXW11 followed by inhibition of the neddylation pathway by MLN4924. SLC16A6 is fully stabilized by co-depletion of BTRC and FBXW11 without notable stability increase by neddylation inhibition (median + upper quartile, n>14,000 cells per condition). **(E)** Graphical illustration for phospho-degron dependent degradation. **(F)** Quantification of SLC16A6-GFP wild-type and phospho-mutants fluorescence at the plasma membrane (n=160 images per condition; unpaired student t-test was used to compare phospho-mutants against wildtype). Representative images of the S240A and T245E mutant are highlighted below the quantification plot. Lower and upper hinges of box plots correspond to the 25th and 75th percentiles, respectively. Lower and upper whiskers extend from the hinge to the smallest or largest value no further than the 1.5× interquartile range from the hinge, respectively. Black line represents the mean and dots represent outliers. **(G)** Protein stability assay of SLC16A6^WT^ and SLC16A6 phospho-mutants in combination with RNAi treatment of adaptor proteins BTRC and FBXW11 (n=4). **(H)** Spectral counts of SCF-complex subunits quantified in AP-MS experiments of SLC16A6 phospho-mutants versus SLC16A6^WT^. Data are represented as mean spectral counts ± SD. Points represent SPC for four MS-injections (n=2 biologically independent replicates with n=2 technical injections).


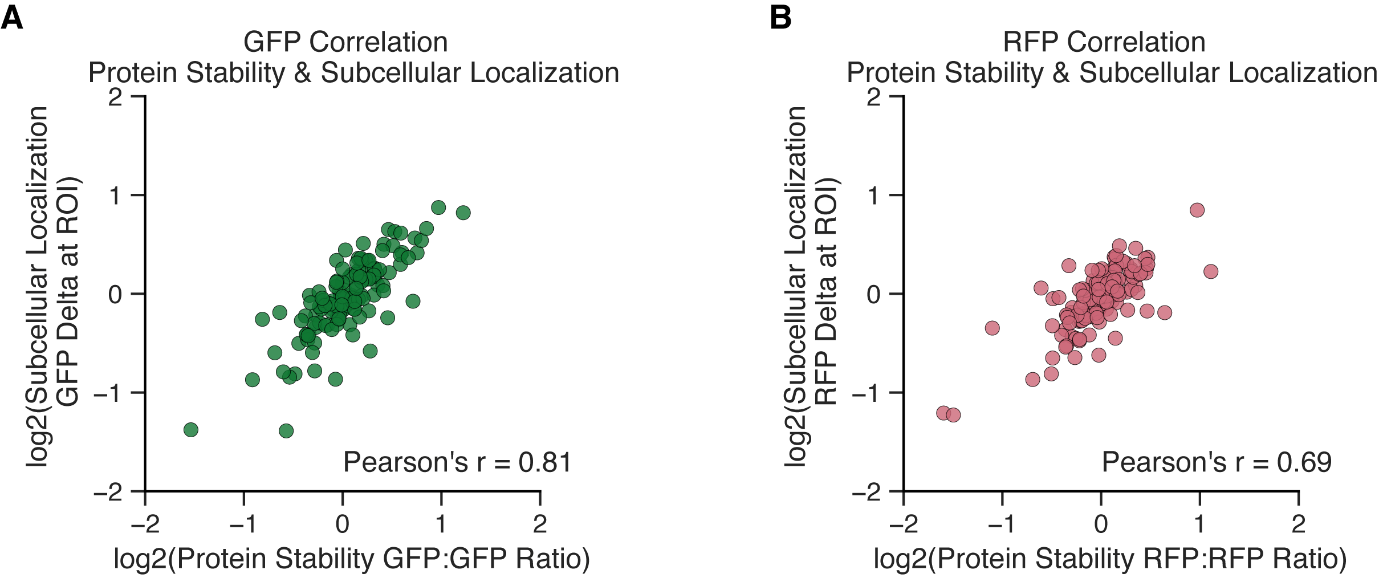


Appendix Figure S13. Correlation of relative fluorescence changes after depletion of SLC interactors in protein stability and subcellular localization assays (Fig. 4, Fig. 6). **(A)** GFP intensity changes after depletion or overexpression of an SLC interactor compared to control treatment measured at the cytometer (horizontal axis) and at the microscope at the region of interest (ROI) marked by RFP (vertical axis) are plotted. The relative GFP intensity changes correlate well (Pearson correlation coefficient R=0.81166, p=7.48938e^-33^). **(B)** RFP intensity changes after depletion or overexpression of an SLC interactor compared to control treatment measured at the cytometer (horizontal axis) and at the microscope at the region of interest (ROI) marked by RFP (vertical axis) are plotted. The relative RFP intensity changes correlate well (Pearson correlation coefficient R=0.6914, p=1.65633e^-20^).

**
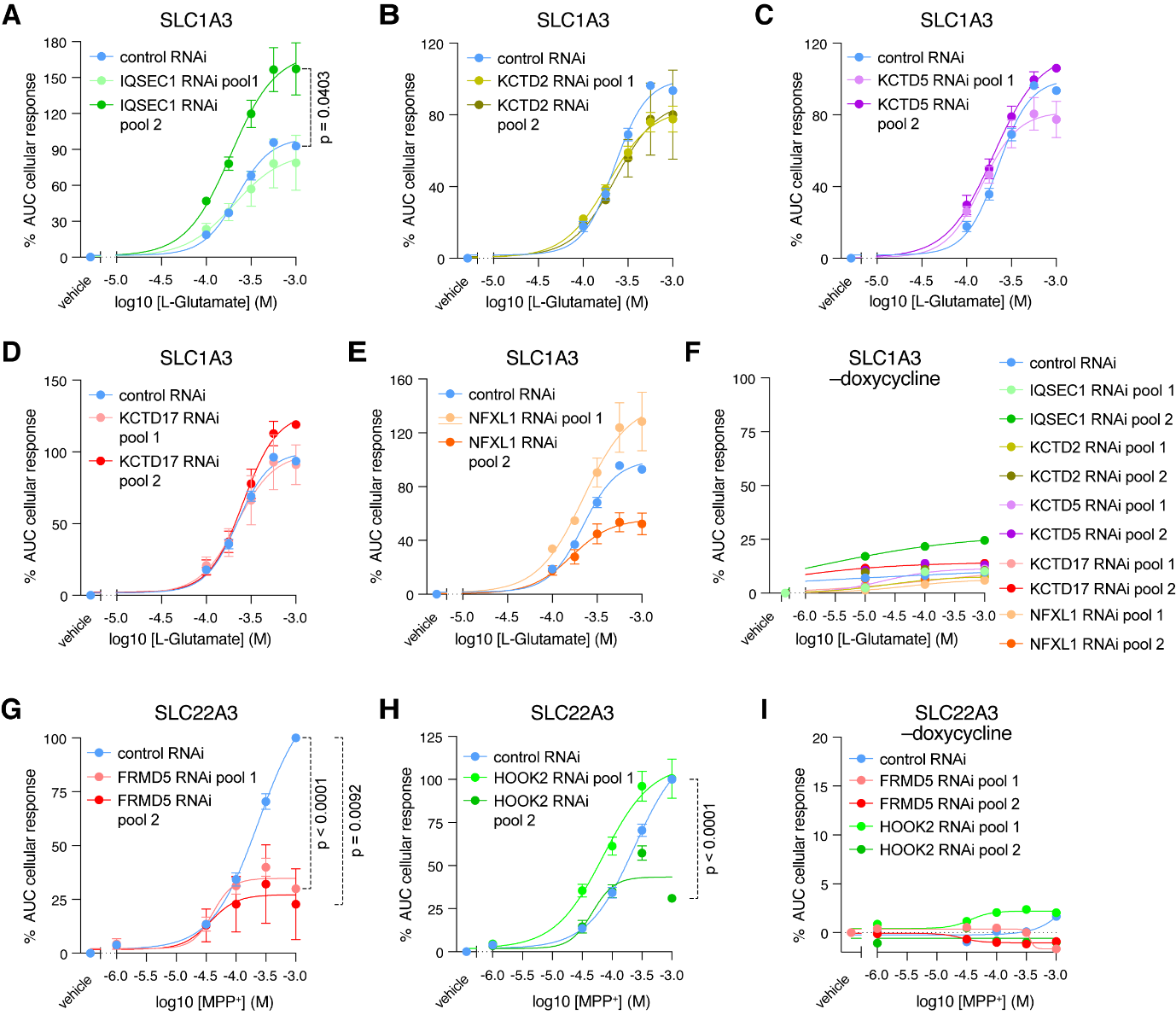
**

Appendix Figure S14. Transporter function of SLC1A3 and SLC22A3 is affected by the depletion of distinct interactors. **(A–E)** HEK 293 Jump In T-REx cells expressing SLC1A3-SH (RESOLUTE Cell line ID CE029W-F) were treated with DsiRNA pools against its interactors **(A)** IQSEC1, **(B)** KCTD2, **(C)** KCTD5, **(D)** KCTD17 or **(E)** NFXL1 for 2 days before dox induction for 24 hours. Cells were stimulated with increasing concentrations of L-Glutamate and transport activity was detected as phenotypic impedance measured by an xCELLigence instrument (Sijben *et al*, 2022). Data are shown as background corrected mean area under the curve (AUC) quantification of three experiments (each performed in duplicate) relative to the negative control RNAi ±SEM. p-values were calculated using unpaired t-test. Significant differences are indicated compared to control RNAi. **(F)** Cells were processed as in (A–E) without the addition of doxycycline to measure background response to the RNAi. Data are shown as background corrected mean AUC quantification of 3 experiments (in duplicate) relative to the control RNAi ± SEM. **(G–I)** HEK 293 Jump In T-REx cells expressing SLC22A3-SH (RESOLUTE Cell line ID CE028P-M) were treated with DsiRNA pools against its interactors **(G)** FRMD5 or **(H)** HOOK2 for 2 days before dox induction for 24 hours. Cells were stimulated with increasing concentrations of neurotoxin MPP^+^ and response was monitored for 2 hours by xCELLigence (Mocking *et al*, 2022). Shown are background corrected mean AUC quantifications of 3 experiments (each performed in duplicate) relative to the control RNAi ±SEM. p-values were calculated using unpaired t-test. Significant differences are indicated compared to control RNAi.


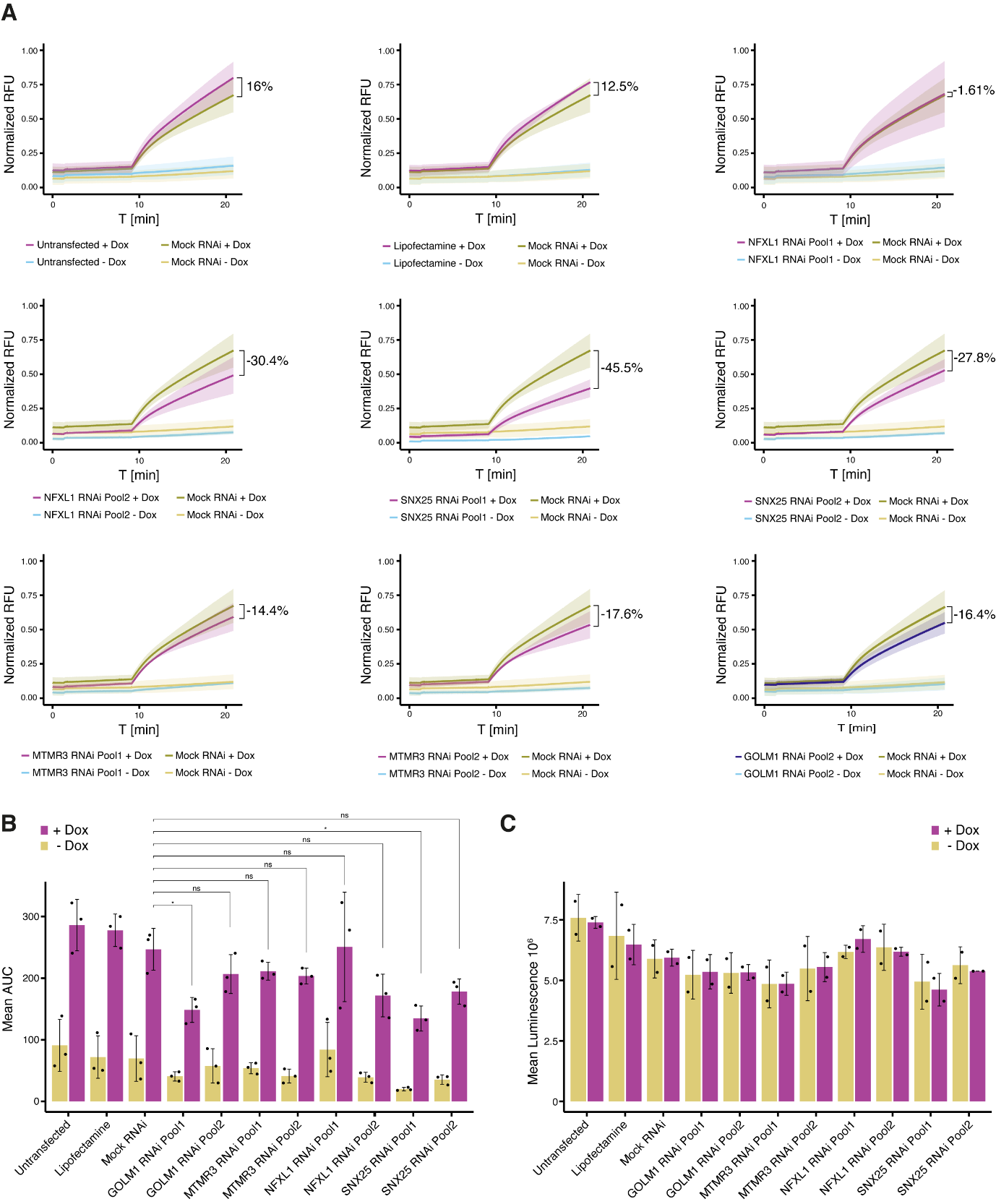


Appendix Figure S15. Transporter assay results for SLC39A8 after RNAi of selected interaction partners. **(A)** Relative fluorescence unit (RFU, excitation wavelength of 480 nm and an emission of 540 nm) curve to measure the transport activity of SLC39A8 after RNAi against GOLM1, MTMR3, NFXL1 and SNX25. For each target two RNAi pools are shown against a mock RNAi treated control. Each condition was tested with and without the induction of SLC39A8. The traces represent the mean min-max normalized RFU values across replicates and the curve shades indicate the +/- SD for each time point (n=3 biologically independent replicates, with n=8 technical replicates/wells). **(B)** Mean area under the curve (AUC) per each RNAi pool for the dox induced (purple) vs. uninduced condition (sand). The mean AUC (bars) were calculated from the normalized RFU traces of all replicates. Overlayed black points represent the biological replicates (n=3 biologically independent replicates, with n = 8 technical replicates/wells). Significance of changes in transport activity for dox induced conditions was tested against control RNAi pool (negative control) using an unpaired two-tailed Student t-test. A significant change was only found for GOLM1 RNAi and SNX25 RNAi (“*” p-value < 0.05). **(C)** Cell viability assay (Cell Titer-Glo) of HEK 293 WT OE cell lines treated with RNAi pools targeting prey proteins. No significant change was found with an unpaired Student t-test (n=2 biologically independent replicates).


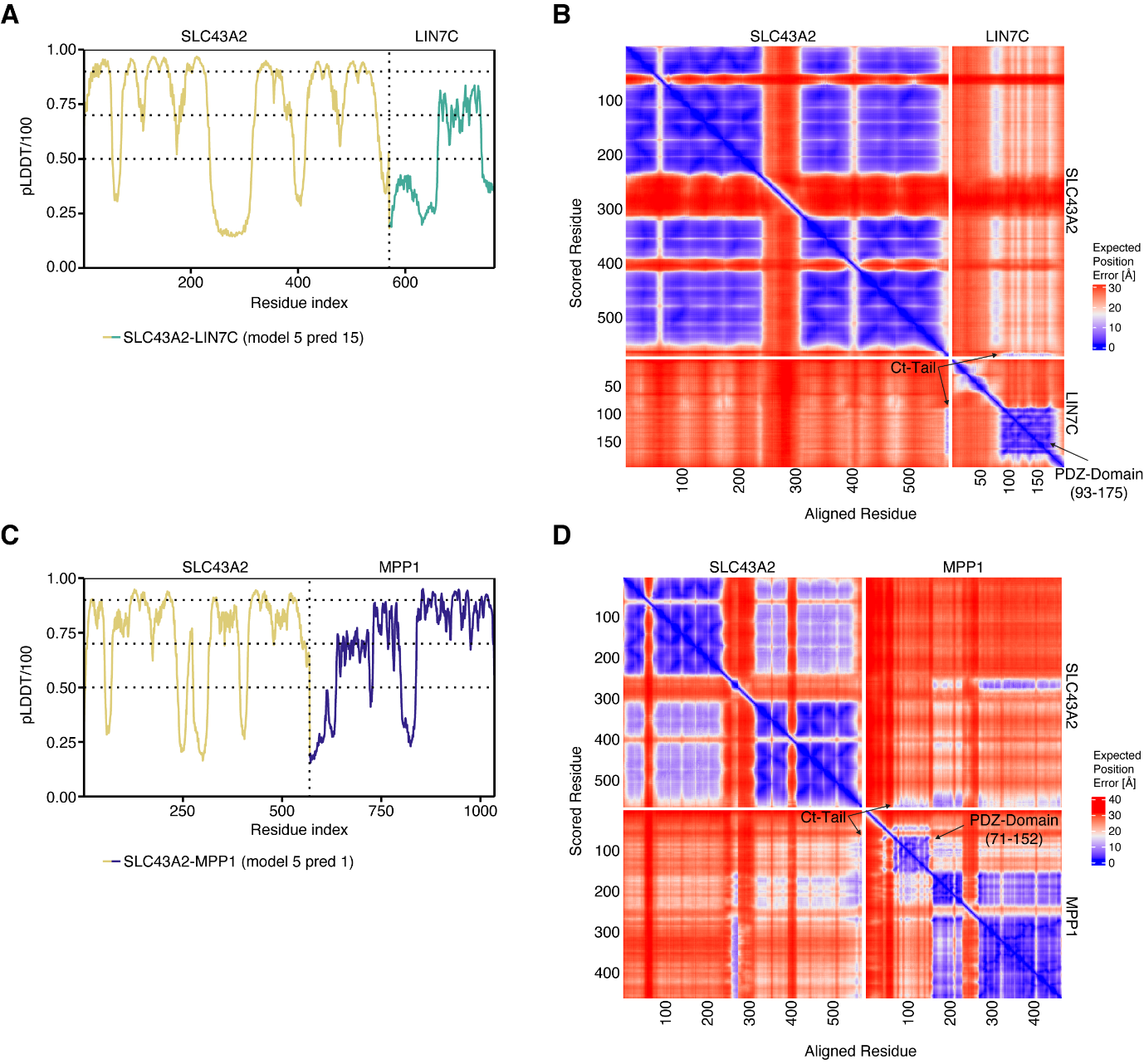


Appendix Figure S16. Prediction of the structure of SLC43A2 interactions with LIN7C and MPP1 by AlphaFold multimer**. (A)** AlphaFold multimer confidence in pLDDT/100 for best ranked model of the SLC43A2-LIN7C interaction. **(B)** Inter PAE heatmap for the best ranked model of the SLC43A2-LIN7C interaction. The interaction of the CT-tail of SLC43A2 and the PDZ domain of LIN7C are indicated in the map. **(C)** AlphaFold multimer confidence in pLDDT/100 for best ranked model of the SLC43A2-MPP1 interaction. **(D)** Inter PAE heatmap for the best ranked model of the SLC43A2-MPP1 interaction. The interaction of the CT-tail of SLC43A2 and the PDZ domain of MPP1 are indicated in the map.


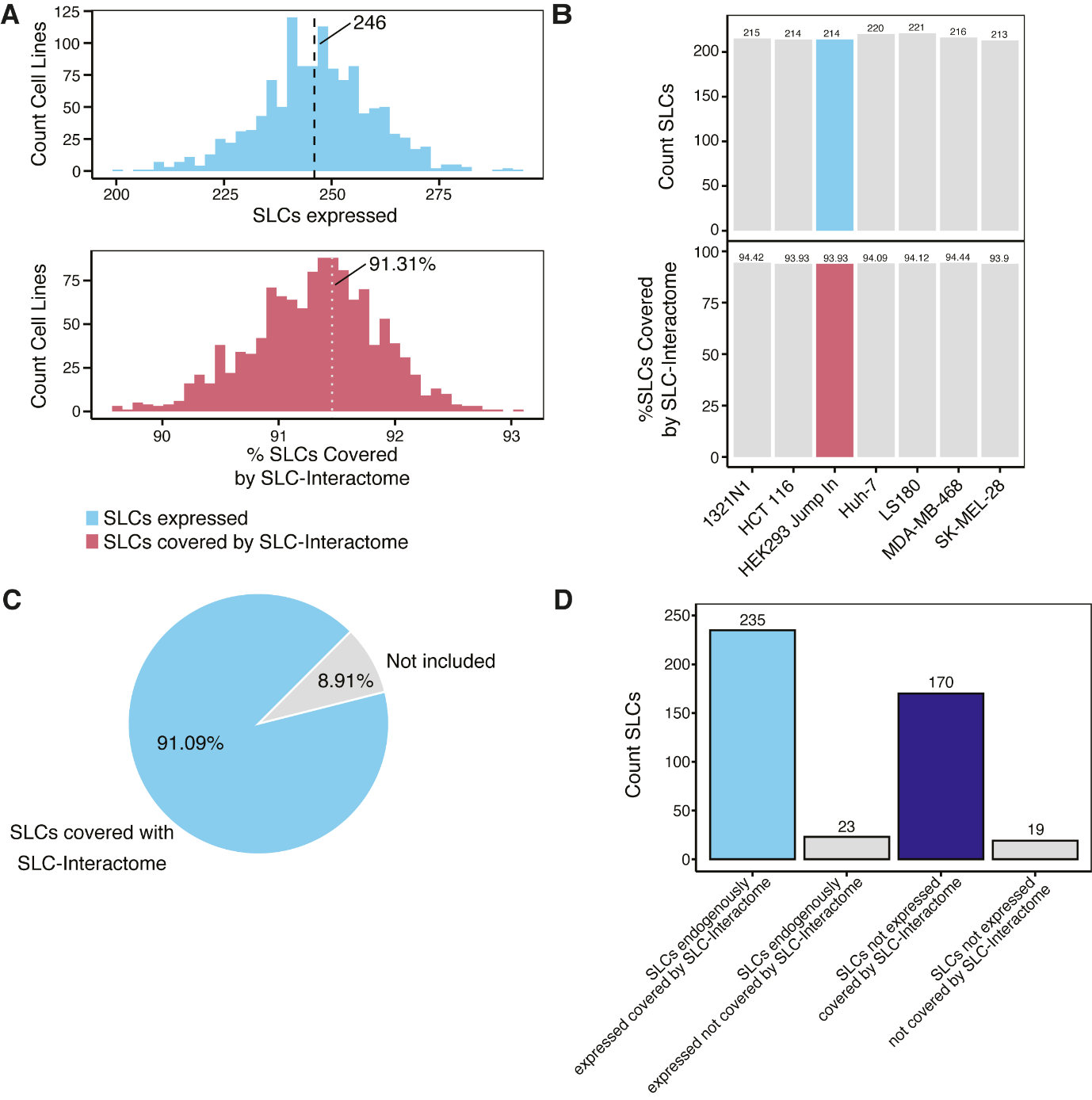


Appendix Figure S17. Coverage of endogenously expressed SLCs in transcriptome, full proteome profiling of HEK 293 Jump-In compared to SLC-interactome. **(A)** Expression of 447 SLCs (Meixner *et al*, 2020) across 1206 human cell lines reported by Human protein atlas (Uhlén *et al*, 2015). A gene was considered to be expressed if the normalized TPM was larger or equal to 1. Expression of SLCs across cell lines is shown in blue. HEK 293WT is ranked at position 613 across all the cell lines and has 246 express SLCs (HEK 293 WT indicated black dashed line). The median and mean of all cell lines were 246 and246.5 SLCs respectively. Coverage of SLCs (in red) used as bait in this study, HEK 293WT is with 91.46% (dotted grey line) close to the mean (91.31%) and median (91.35%). **(B)** SLCs quantified by expression proteomics across seven human cell lines and the coverage in percentage of SLC bait proteins included in the study. HEK 293 Jump-In cell line (blue) has 214 SLCs quantified by proteomics and 93.93% of all SLCs endogenously expressed and covered by proteomics are included in the interaction study. **(C)** SLCs expressed and covered within our study (blue) and SLCs expressed in HEK 293 Jump-In but not covered (grey). Within the SLC-interactome 91.09% of SLCs expressed with more than 1 TPM in HEK 293 Jump In are covered. **(D)** SLCs endogenously expressed in HEK 293 Jump In and reported in the interactome (blue), endogenously expressed but not part of the SLC-interactome dataset (grey), SLC as baits within the SLC-interactome but not expressed in HEK 293 Jump-In (dark blue) and the SLCs which are not part of SLC-interactome and also not expressed in HEK 293 Jump In (grey). With 89.95% coverage of the non-expressed, the two groups have comparable coverage.


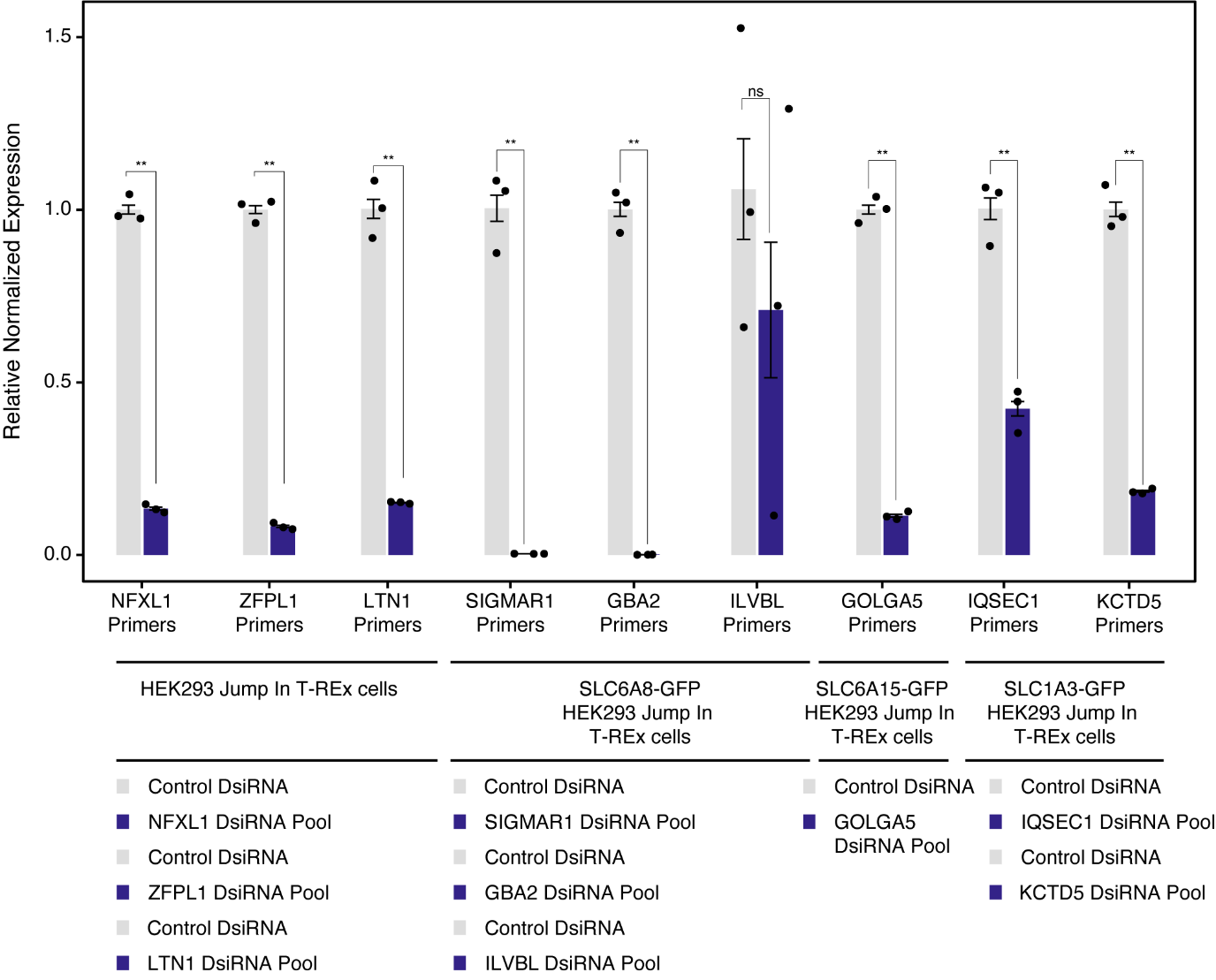


Appendix Figure S18. Validation of 9 DsiRNA pools used for validation of SLC-protein interactions. Relative normalized expression for genes targeted by DsiRNA pools. The ∆Cq was calculated against the housekeeping gene HPRT1 for target and control DsiRNA samples. The 2^-∆∆Cq^ (delta-delta cycle quantification threshold) per target gene (blue bars) were derived against control DsiRNA samples (gray). Bars represent the average across replicates (n=3) and error bars represent +/- SEM of relative normalized expression. Data were analyzed with unpaired Student's t-tests; significant hits are marked with asterisks (‘**’) representing a p-value ≤ 0.01. Validation of RNAi pools were conducted in different cell lines indicated below each graph. Samples expressing GFP-tagged SLCs were processed alongside protein stability and cytometry experiments (**Dataset EV7**) and expression was induced for 24 h with doxycycline.


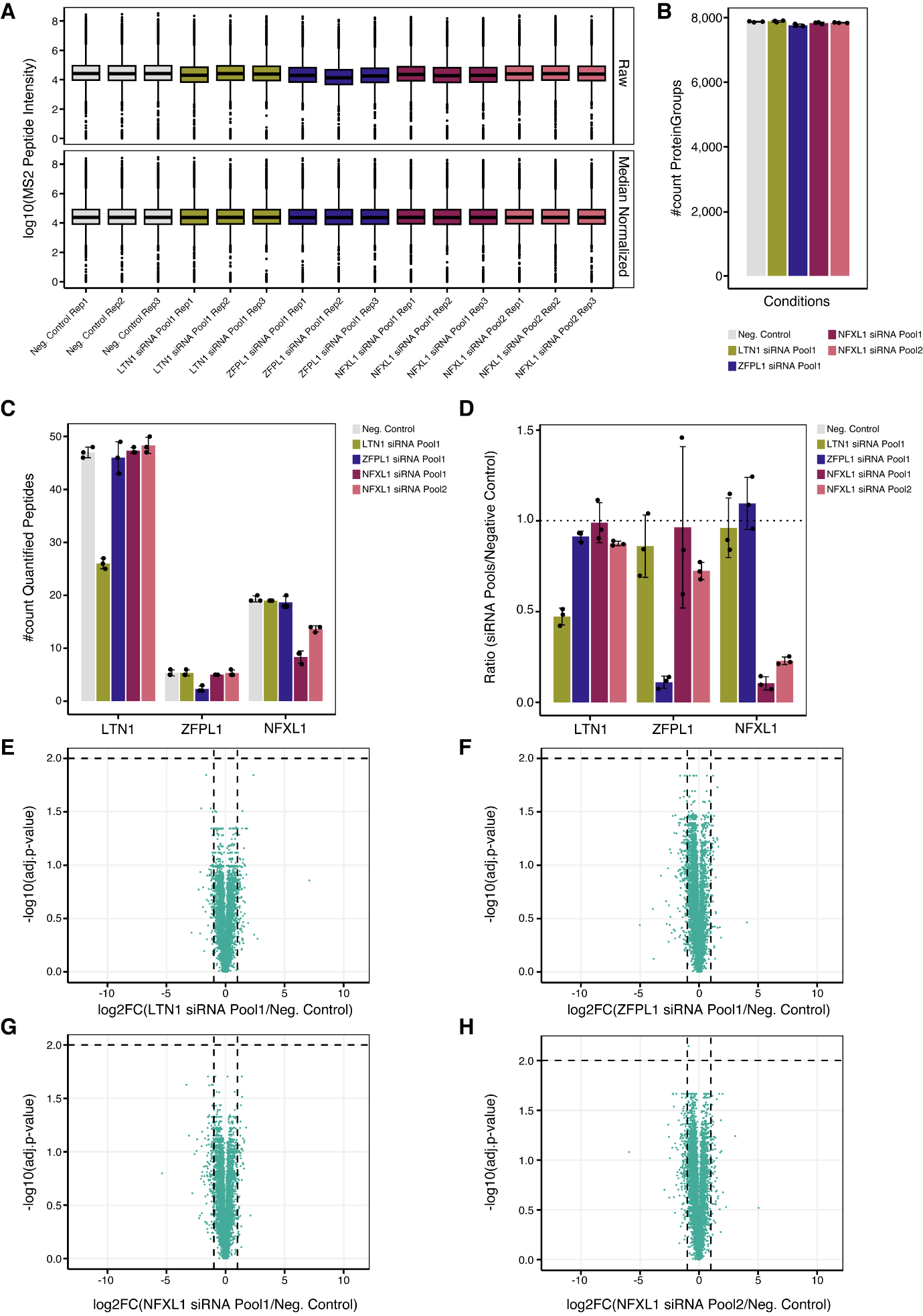


Appendix Figure S19. Assessment of RNAi efficiency targeting three SLC interaction partners by full proteome profiling in HEK 293 Jump In T-REx cells. **(A)** Distribution of peptide intensities before (upper panel) and after median normalization (lower panel). Lower and upper hinges of box plots correspond to the 25^th^ and 75^th^ percentiles, respectively. Lower and upper whiskers extend from the hinge to the smallest or largest value no further than the 1.5× interquartile range from the hinge, respectively. Black line represents the median and the black dots represent outliers. The experiment was conducted with n=3 biological replicates. **(B)** Quantified proteins per RNAi sample condition. The bars indicate the average quantified proteins whereas the error bars are +/- SD of the mean. Black dots represent the count of quantified proteins per sample (n=3 biological replicates). Colors indicate different siRNA pools. On the x-axis the target genes of the siRNA pools are indicated. **(C)** Peptides of the target genes LTN1 (olive), ZFPL1 (dark blue) and NFXL1 (siRNA pool 1: dark red; siRNA pool 2: red) quantified within each sample and the negative siRNA control (grey). **(D)** For each targeted gene, the ratios against the negative control RNAi samples were derived. The x-axis shows the ratio against negative control siRNA within each of the different samples (indicated by color, see legend), whereas the y-axis shows the ratio normalized to the negative control. Within RNAi target samples the corresponding protein abundance was reduced from 50% to 90% relative to the control. **(E)** Differential enrichment of proteins in LTN1 RNAi treated samples against negative controls. The x-axis represents the log2FC of each protein against the signal in the control. The y-axis shows the -log10 of the adjusted p-value (unpaired Student t-test, BH correction). As thresholds for significantly enriched proteins a log2FC ≥ 2 and an adjusted p-value ≤ 0.01 were used (dotted lines). Proteins are shown in teal. **(F)** Differential enrichment of proteins in ZFPL1 DsiRNA treated samples against negative controls. Thresholds were set as in panel (E). **(G)** and **(H)** show differential enrichment of proteins in NFXL1 DsiRNA treated samples against negative controls for DsiRNA pool 1 and pool 2 targeting NFXL1. Thresholds were set as in panel (E).

# References

Meixner E, Goldmann U, Sedlyarov V, Scorzoni S, Rebsamen M, Girardi E & Superti-Furga G (2020) A substrate-based ontology for human solute carriers. *Mol Syst Biol* 16: e9652

Mocking TAM, Sijben HJ, Vermeulen YW, IJzerman AP & Heitman LH (2022) MPP+-Induced Changes in Cellular Impedance as a Measure for Organic Cation Transporter (SLC22A1-3) Activity and Inhibition. *Int J Mol Sci* 23

Sijben HJ, Dall’ Acqua L, Liu R, Jarret A, Christodoulaki E, Onstein S, Wolf G, Verburgt SJ, Le Dévédec SE, Wiedmer T, *et al* (2022) Impedance-Based Phenotypic Readout of Transporter Function: A Case for Glutamate Transporters. *Front Pharmacol* 13: 872335

Uhlén M, Fagerberg L, Hallström BM, Lindskog C, Oksvold P, Mardinoglu A, Sivertsson Å, Kampf C, Sjöstedt E, Asplund A, *et al* (2015) Tissue-based map of the human proteome. *Science* 347: 1260419
