# Supplementary figures and images for: The solute carrier superfamily interactome
Source: Mol Syst Biol. 2025 May 12;21(6):632–75. doi: 10.1038/s44320-025-00109-1 (PMC12130317; doi:10.1038/s44320-025-00109-1)

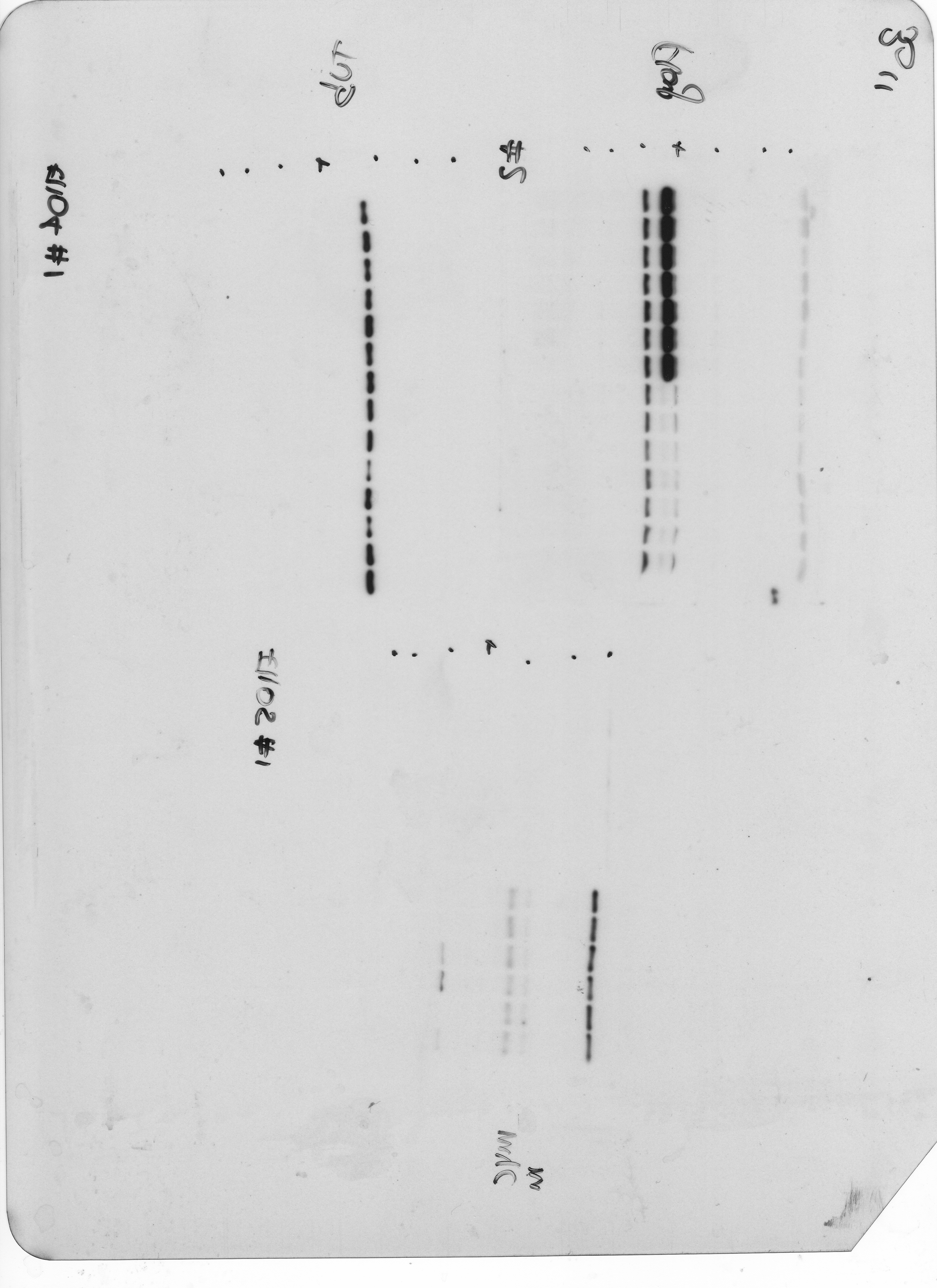

Supplement: Supplementary file 23 — Source Data Fig. EV4C [file 44320_2025_109_MOESM23_ESM.zip › Source_data/EV4C/Fig_EV4_1_KLHL36-FLAG_alpha_tublin_short_exposure_only_left_side_E1104.jpg]

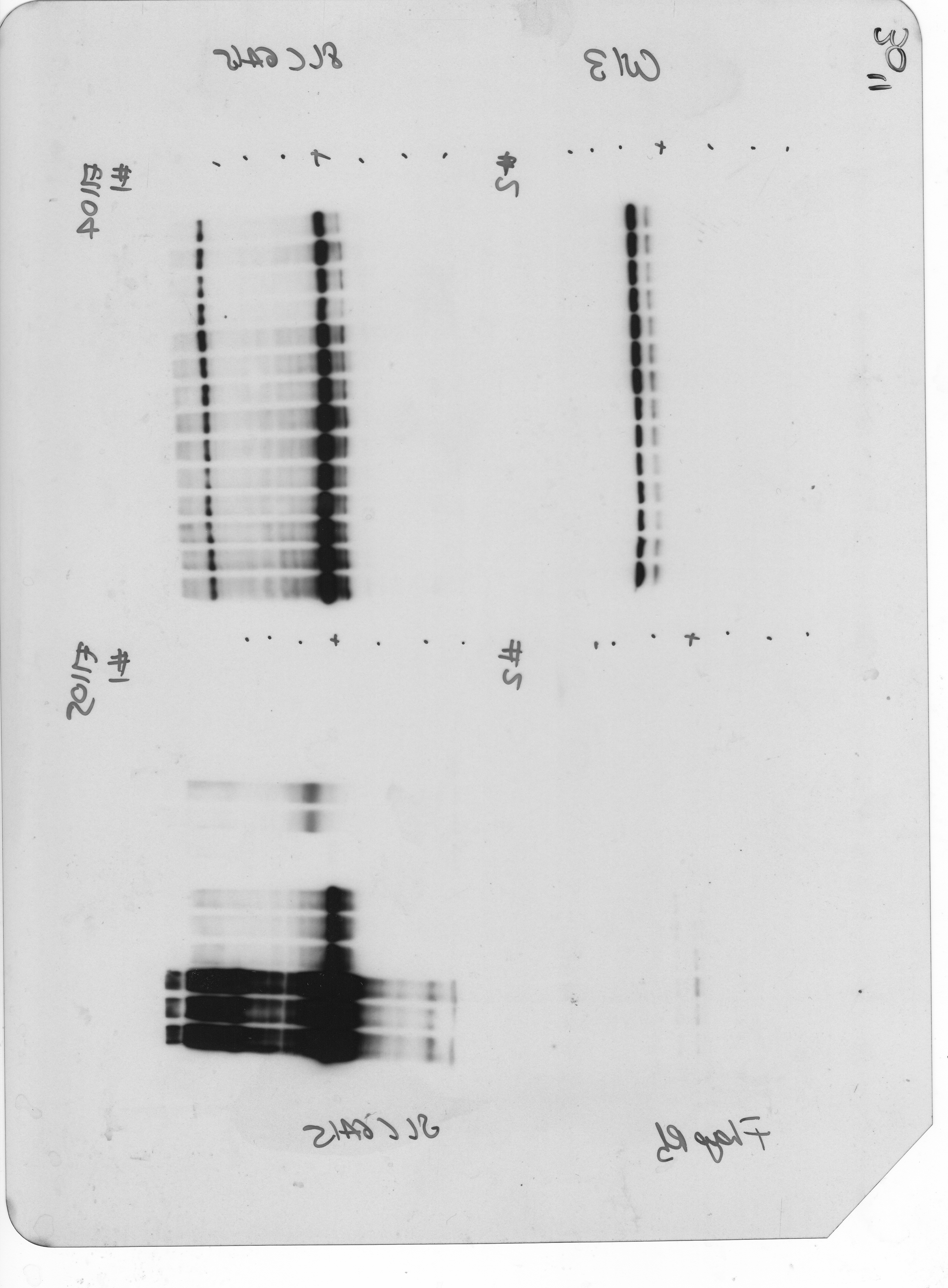

Supplement: Supplementary file 23 — Source Data Fig. EV4C [file 44320_2025_109_MOESM23_ESM.zip › Source_data/EV4C/Fig_EV4_1_SLC6A8_Cul3_long_exposure_only_left_side_E1104.jpg]

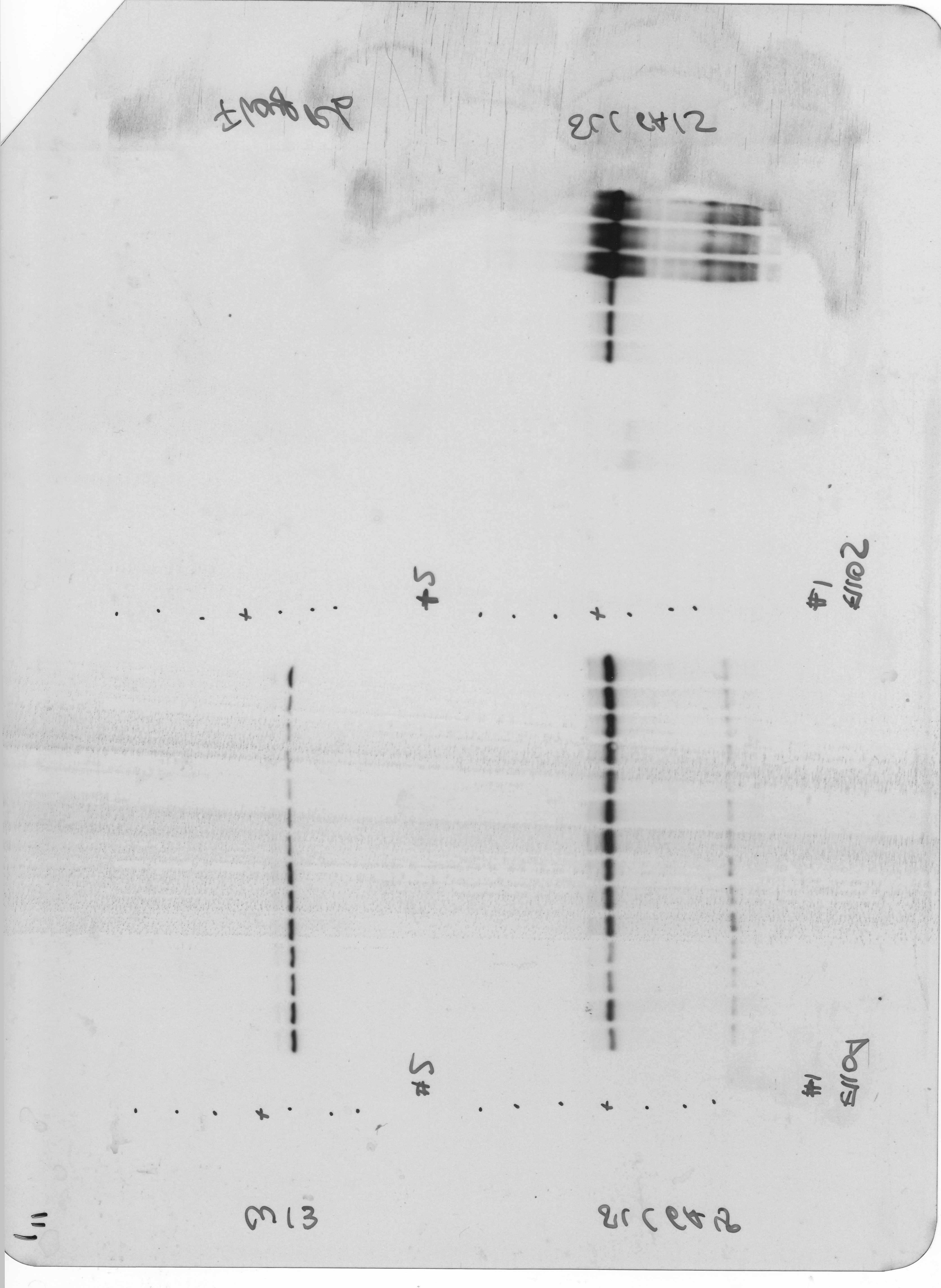

Supplement: Supplementary file 23 — Source Data Fig. EV4C [file 44320_2025_109_MOESM23_ESM.zip › Source_data/EV4C/Fig_EV4_1_SLC6A8_Cul3_short_exposure_only_left_side_E1104.jpg]
