# Supplementary material for: The solute carrier superfamily interactome
Source: Mol Syst Biol. 2025 May 12;21(6):632–75. doi: 10.1038/s44320-025-00109-1 (PMC12130317; doi:10.1038/s44320-025-00109-1)
Supplement: Supplementary file 24 — Expanded View Figures [file 44320_2025_109_MOESM24_ESM.pdf]

# Expanded View Figures

**Figure EV1. Characterization of SLC-protein interactions, SLCs as background and SLCs in complexes.**

(**A**) SLCs and PPIs reported across protein interaction databases. (**B**) Overlap of SLCs and PPIs across protein interaction databases. (**C**) Reported PPIs across the PPI library were plotted against frequency of identification in the CRAPome database. 127 SLCs were reported in the PPI library and were also part of CRAPome (CRAPome frequency >20% in violet, <20% in gray). (**D**) Comparison of the share of PPIs reported in the prey role for SLCs in BioGRID with a CRAPome frequency >20% ($n = 11$) against the share of PPIs reported in the prey role for SLCs with a CRAPome frequency <20% ($n = 75$). Data are presented as mean ± SD. The mean of the two groups showed a significant difference (two-sample t test, $P$ value: 0.005725). In the figure panel, $P$ values below 0.01 are indicated with "**". (**E**) Count of protein complexes (upper part) and protein complex subunits (lower part) included in CORUM. Subunits/ complexes were grouped into SLC, TM protein and no-TM protein and were counted across all reported protein complexes, taking into consideration multiple occurrences of subunits. (**F**) GALNT2 signal distribution across the SLC interactome. The left panel displays log2 transformed spectral counts for each SLC AP-MS experiment, grouped by scored interactions ($n = 21$, blue) and background/non-interacting ($n = 230$, gray). Lower and upper hinges of box plots correspond to the 25th and 75th percentiles, respectively. Lower and upper whiskers extend from the hinge to the smallest or largest value no further than the 1.5× interquartile range from the hinge, respectively. Black line represents the median log2 spectral count signal, and the black dots represent the signal per measurement. The right side shows the frequency of GALNT2 identification, scoring, and background in percentages. (**G**) The upper bar chart shows all SLCs for which GALNT2 was scored within the SLC interactome (log2FC against GFP, threshold of log2FC > 1). The lower section of the bar chart reports the confidence scores of predicted GALNT2-SLC complexes (high confidence, pDockQ threshold of >0.5 dashed black line, medium confidence, pDockQ threshold of >0.25 represented by a dashed gray line). A comparison against randomly sampled SLC-GALNT2 interaction (see "Methods" for details) showed, a significant difference between models of the interactions covered in the SLC interactome ($n = 21$) and the control set ($n = 21$; unpaired Student $t$ test, $P$ value = 0.00695). In the figure panel, $P$ values below 0.01 are indicated with "**". Lower and upper hinges of box plots correspond to the 25th and 75th percentiles, respectively. Lower and upper whiskers extend from the hinge to the smallest or largest value no further than the 1.5× interquartile range from the hinge, respectively. Black line represents the median and the black dots represent scores per complex. (**H**) The AlphaFold model of SLC30A1-GALNT2 is shown on the right side.

 © The Author(s)

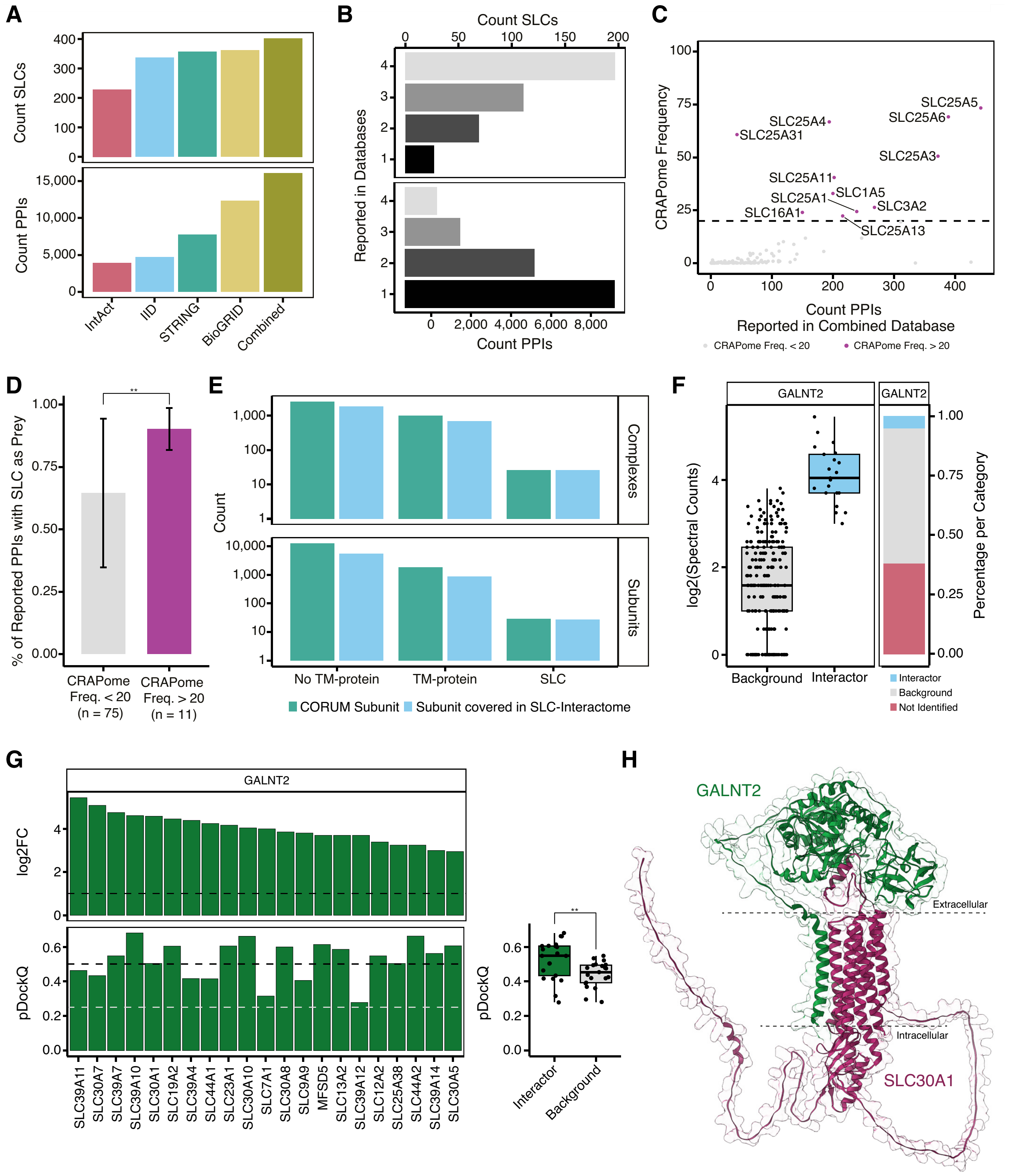

A
Count SLCs
Count PPIs
IntAct
IID
STRING
BioGRID
Combined
B
Count SLCs
Reported in Databases
Count PPIs
C
CRAPome Frequency
SLC25A5
SLC25A6
SLC25A4
SLC25A31
SLC25A3
SLC25A11
SLC1A5
SLC25A1
SLC3A2
SLC16A1
SLC25A13
Count PPIs
Reported in Combined Database
CRAPome Freq. < 20
CRAPome Freq. > 20
D
% of Reported PPIs with SLC as Prey
CRAPome Freq. < 20 (n = 75)
CRAPome Freq. > 20 (n = 11)
E
Count
Complexes
Subunits
No TM-protein
TM-protein
SLC
CORUM Subunit
Subunit covered in SLC-Interactome
F
GALNT2
log2(Spectral Counts)
Background
Interactor
Percentage per Category
Interactor
Background
Not Identified
G
GALNT2
log2FC
pDockQ
SLC39A11
SLC30A7
SLC39A7
SLC39A10
SLC30A1
SLC19A2
SLC39A4
SLC44A1
SLC23A1
SLC30A10
SLC7A1
SLC30A8
SLC9A9
MFSD5
SLC13A2
SLC39A12
SLC12A2
SLC25A38
SLC44A2
SLC39A14
SLC30A5
Interactor
Background
H
GALNT2
Extracellular
Intracellular
SLC30A1

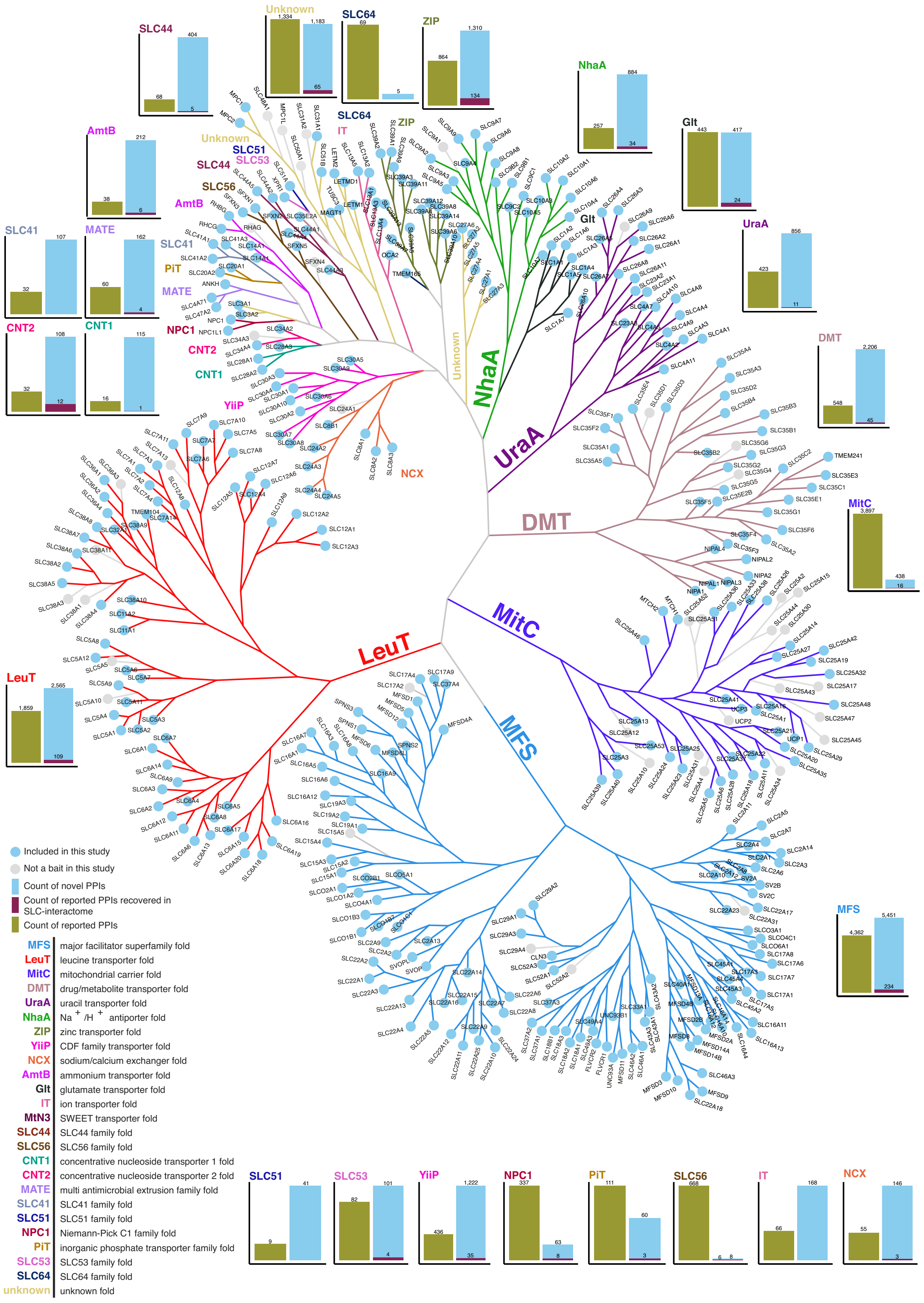

MFS
LeuT
MitC
DMT
UraA
NhaA
Glt
ZIP
NCX
YiiP
CNT1
CNT2
NPC1
MATE
PiT
SLC41
AmtB
SLC56
SLC44
SLC51
SLC53
SLC64
IT
Unknown
Included in this study
Not a bait in this study
Count of novel PPIs
Count of reported PPIs recovered in SLC-interactome
Count of reported PPIs
MFS major facilitator superfamily fold
LeuT leucine transporter fold
MitC mitochondrial carrier fold
DMT drug/metabolite transporter fold
UraA uracil transporter fold
NhaA Na + /H + antiporter fold
ZIP zinc transporter fold
YiiP CDF family transporter fold
NCX sodium/calcium exchanger fold
AmtB ammonium transporter fold
Glt glutamate transporter fold
IT ion transporter fold
MtN3 SWEET transporter fold
SLC44 SLC44 family fold
SLC56 SLC56 family fold
CNT1 concentrative nucleoside transporter 1 fold
CNT2 concentrative nucleoside transporter 2 fold
MATE multi antimicrobial extrusion family fold
SLC41 SLC41 family fold
SLC51 SLC51 family fold
NPC1 Niemann-Pick C1 family fold
PiT inorganic phosphate transporter family fold
SLC53 SLC53 family fold
SLC64 SLC64 family fold
unknown unknown fold


 © The Author(s)

**Figure EV2. Novel versus known protein interactions for each clade of the structural and evolutionary-based SLCome.**

For the construction of the phyologenetic tree the distance matrix of a previous classification of the SLC superfamily based on structural models was used (Ferrada and Superti-Furga, 2022). Each of the 25 distinct structural clades are represented in a different color. For each clade, the count of novel PPIs (light blue), literature mined PPIs (olive), and the shared PPIs (dark red) are reported. The 405 SLCs included in the study are colored light blue and all SLCs which were not included in the SLC interactome are colored gray.

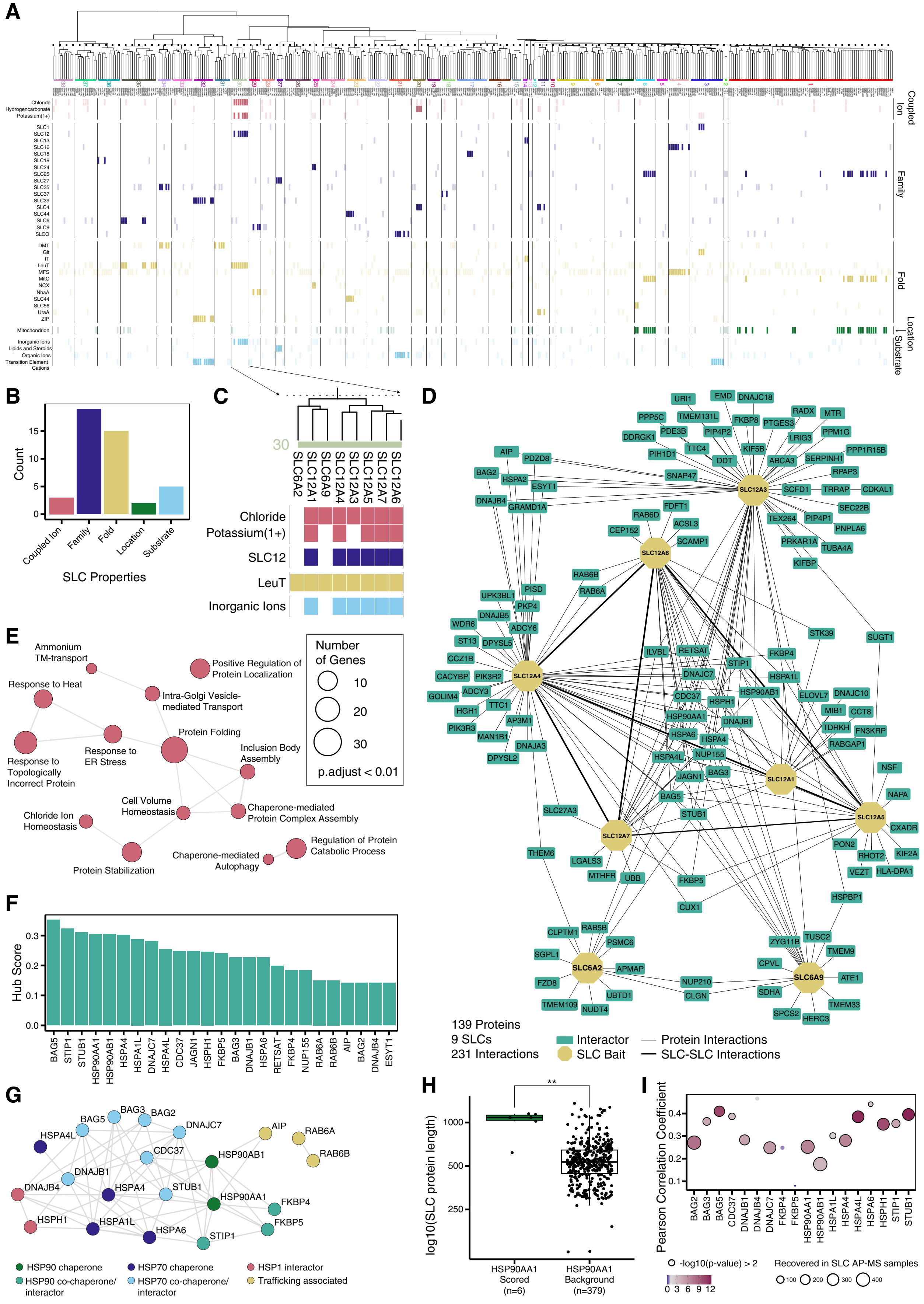
A
Coupled Ion
Family
Fold
Location
Substrate
B
Count
Coupled Ion
Family
Fold
Location
Substrate
SLC Properties
C
30
SLC6A2
SLC12A1
SLC6A9
SLC12A4
SLC12A3
SLC12A5
SLC12A7
SLC12A6
Chloride
Potassium(1+)
SLC12
LeuT
Inorganic Ions
D
139 Proteins
9 SLCs
231 Interactions
Interactor
SLC Bait
Protein Interactions
SLC-SLC Interactions
E
Ammonium TM-transport
Positive Regulation of Protein Localization
Response to Heat
Intra-Golgi Vesicle-mediated Transport
Protein Folding
Response to Topologically Incorrect Protein
Response to ER Stress
Inclusion Body Assembly
Cell Volume Homeostasis
Chaperone-mediated Protein Complex Assembly
Chloride Ion Homeostasis
Regulation of Protein Catabolic Process
Chaperone-mediated Autophagy
Protein Stabilization
Number of Genes
10
20
30
p.adjust < 0.01
F
Hub Score
BAG5
STIP1
STUB1
HSP90AA1
HSP90AB1
HSPA4
HSPA1L
DNAJC7
HSPA4L
CDC37
JAGN1
HSPH1
FKBP5
BAG3
DNAJB1
HSPA6
RETSAT
FKBP4
NUP155
RAB6A
RAB6B
AIP
BAG2
DNAJB4
ESYT1
G
HSP90 chaperone
HSP70 chaperone
HSP1 interactor
HSP90 co-chaperone/ interactor
HSP70 co-chaperone/ interactor
Trafficking associated
H
log10(SLC protein length)
HSP90AA1 Scored (n=6)
HSP90AA1 Background (n=379)
I
Pearson Correlation Coefficient
-log10(p-value) > 2
Recovered in SLC AP-MS samples

**Figure EV3. Functional property enrichment analysis showed SLC interactome similarity for SLC12 and SLC6 family members.**

(**A**) Significantly enriched SLC functional properties identified in the interactome SLC clustering analysis (Fisher's test $P < 0.2$). Colors indicate different SLC functional properties. (**B**) Sum of enriched SLC functional properties separated by SLC properties. (**C**) Significantly enriched SLC functional properties for PPI-interactome profile cluster 30 (Fisher's test $P < 0.2$). (**D**) Cluster-specific PPI-network obtained by the SLC baits (yellow octagons) grouped to cluster 30. Interactors are shown in green, and interactions between SLC baits are highlighted with a thick black line. (**E**) GO biological processing terms significantly enriched (Gene ontology overrepresentation test with a hypergeometric model, Benjamini–Hochberg corrected, *P*-adjusted <0.01) obtained by GSEA for interactors present in the specific PPI-network. The obtained terms were converted to a similarity network with GO semantic similarity and further filtered to showcase the least overlapping significant terms (similarity cutoff of 0.7). (**F**) The 25 most connected interactors (hub score) in the cluster 30-specific PPI-network. (**G**) PPI-network retrieved from literature (STRING confidence score >0.4, physical interactions only) for the 25 most connected interactors within cluster 30. Interactors were grouped as HSP90 chaperones (green), HSP90 co-chaperones/interactors (teal), HSP70 chaperones (blue), HSP70 co-chaperones/interactors (cyan), HSP1 interactors (red) or trafficking associated proteins (yellow). The four interactors not connected were removed from the PPI-network. (**H**) Distribution of protein length of SLCs interacting with HSP90AA1 ($n = 6$) and SLCs for which HSP90AA1 was found in the background ($n = 379$). Comparison showed a significant difference in the protein length (Student *t* test *P* value = $1.285e^{-08}$). In the figure panel, *P* values below 0.01 are marked with "**". Lower and upper hinges of box plots correspond to the 25th and 75th percentiles, respectively. Lower and upper whiskers extend from the hinge to the smallest or largest value no further than the 1.5× interquartile range from the hinge, respectively. Black line represents the median SLC-protein sequence length, and the black dots represent the sequence length per SLC for which HSP90AA1 was identified. (**I**) Correlation of the chaperone/ chaperone interacting protein abundances with the summed SLC tail length (N-terminal and C-terminal). Significant correlations with a *P* value < 0.01 are indicated with black ring.

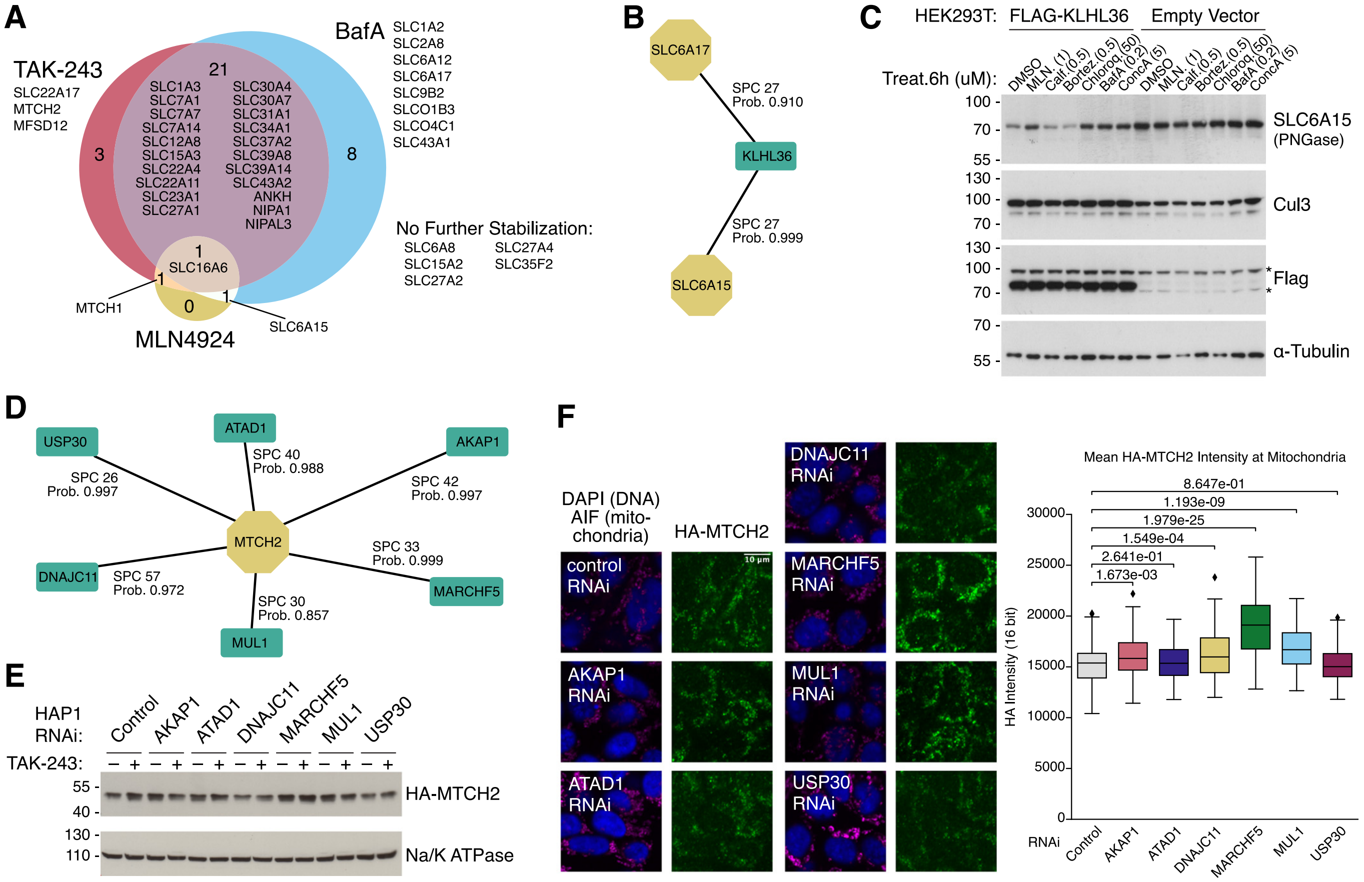


**Figure EV4. SLC levels are regulated by protein stability.**

(**A**) Venn diagram of drug treatment effects on SLC-protein stability. SLCs were considered to be stabilized by the respective drug if the GFP:RFP ratio was increased more than 10% compared to mock treated cells (*P* value < 0.05, independent t test). (**B**) SLC-protein interactions of KLHL36. The protein was not quantified in the background of any other SLC purification. (**C**) Western blot result of endogenous SLC6A15 in HEK 293T cells showing SLC6A15 degradation after overexpression of FLAG-tagged KLHL36. The samples shown in the blot were derived from the same experiment and gels/blots were processed in parallel. Uncropped images are provided as source data. (**D**) MTCH2 interactome showing 7 distinct interactors that were assessed in detail. (**E**) HAP1 cells expressing endogenously HA-tagged MTCH2 were transfected with RNAi against MTCH2 interactors. Depletion of the E3 ligase proteins MARCHF5 and MUL1 stabilizes MTCH2 levels as strongly as ubiquitination inhibition by TAK-243. (**F**) Immunofluorescence example images of HA-MTCH2 and quantification at mitochondria as identified by staining for AIF (apoptosis-inducing factor). Over 10,000 cells were imaged per condition; unpaired t test was used to compare treatments (*n* = 112 images per sample). Lower and upper hinges of box plots correspond to the 25th and 75th percentiles, respectively. Lower and upper whiskers extend from the hinge to the smallest or largest value no further than the 1.5× interquartile range from the hinge, respectively. Black line represents the mean and the black dots represent outliers. Source data are available online for this figure.

 © The Author(s)

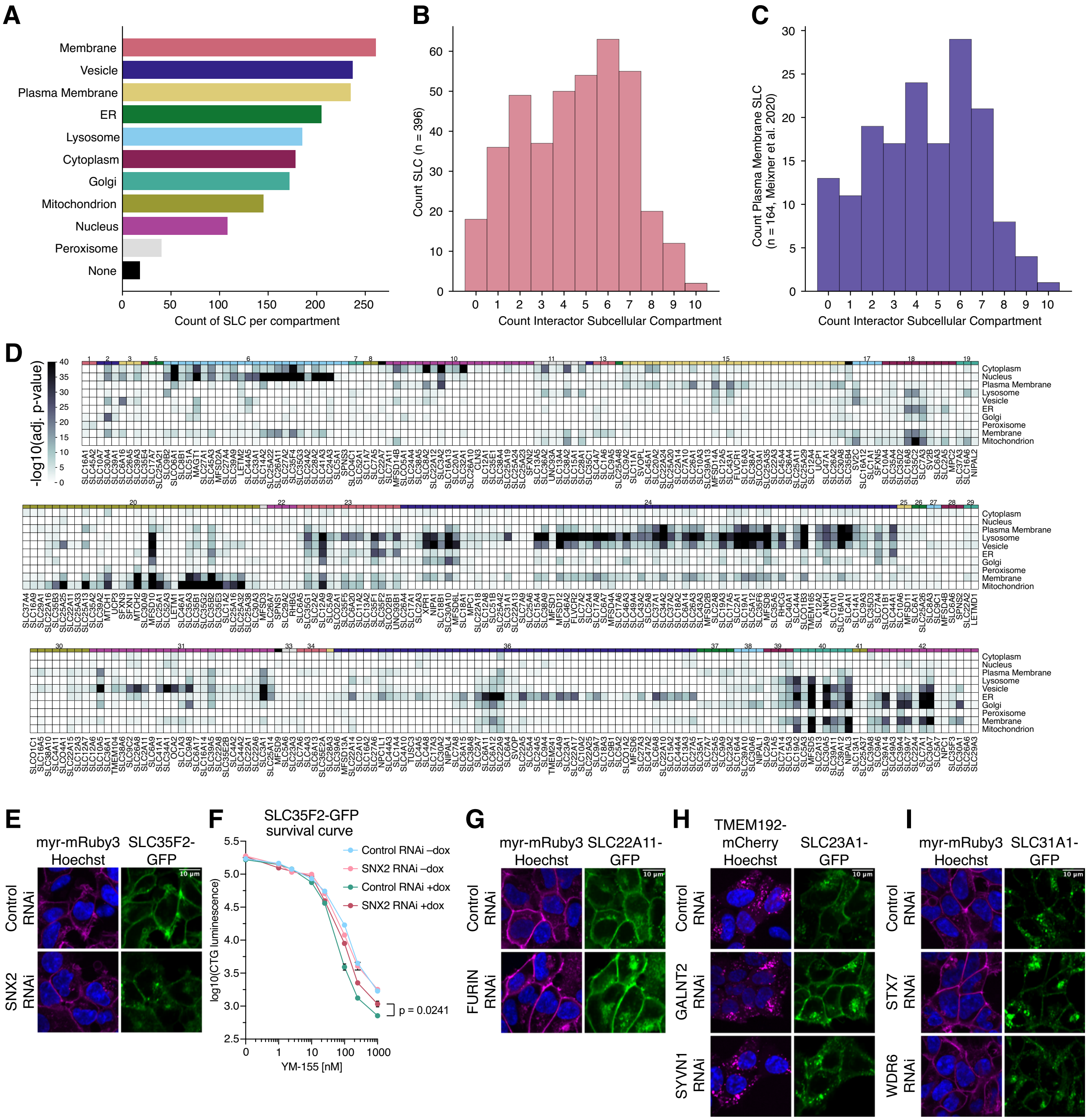
A
Membrane
Vesicle
Plasma Membrane
ER
Lysosome
Cytoplasm
Golgi
Mitochondrion
Nucleus
Peroxisome
None
Count of SLC per compartment
B
Count SLC (n = 396)
Count Interactor Subcellular Compartment
C
Count Plasma Membrane SLC (n = 164, Meixner et al. 2020)
Count Interactor Subcellular Compartment
D
-log10(adj. p-value)
Cytoplasm
Nucleus
Plasma Membrane
Lysosome
Vesicle
ER
Golgi
Peroxisome
Membrane
Mitochondrion
E
myr-mRuby3 Hoechst
SLC35F2-GFP
Control RNAi
SNX2 RNAi
F
SLC35F2-GFP survival curve
Control RNAi –dox
SNX2 RNAi –dox
Control RNAi +dox
SNX2 RNAi +dox
p = 0.0241
log10(CTG luminescence)
YM-155 [nM]
G
myr-mRuby3 Hoechst
SLC22A11-GFP
Control RNAi
FURIN RNAi
H
TMEM192-mCherry Hoechst
SLC23A1-GFP
Control RNAi
GALNT2 RNAi
SYVN1 RNAi
I
myr-mRuby3 Hoechst
SLC31A1-GFP
Control RNAi
STX7 RNAi
WDR6 RNAi
10 μm

© The Author(s) 

**Figure EV5. Proteins affecting trafficking and subcellular localization of SLCs.**

(**A**) GSEA of cellular compartments was performed for each SLC interactome and the resulting GO terms were hierarchically assigned to 10 main compartments (*P* value cutoff 0.05; see "Methods" for details). (**B**) A majority of SLC interactomes contain interaction partners from several subcellular compartments (mean: 4.5 compartments per SLC). (**C**) This notion also holds true for a subset of SLCs described previously as uniquely plasma membrane-associated (mean: 4.3 compartments). (**D**) The results from the interactome–compartment enrichment analysis were used to cluster SLCs. Strong clusters can be identified for some mitochondria baits (e.g., MTCH2), lysosomal baits (e.g., MFSD12) or Golgi baits (e.g., SLC30A5). (**E**) Representative images of plasma membrane- and Golgi-associated SLC35F2 after RNAi (see Fig. 6D for a quantification of the effect; size bar, 10 μm for all images). (**F**) SNX2 depletion attenuates SLC35F2-mediated import of YM-155. After 48 h RNAi, SLC35F2-GFP expression was induced using doxycycline and cells were subjected to YM-155 at the indicated concentrations for 24 h. Cell viability was measured using CellTiter-Glo luminescence ($n = 3$, mean ± SEM). Induced conditions at 1 μM YM-155 were compared using unpaired *t* test. (**G**) Representative images of plasma membrane- and Golgi-associated SLC22A11-GFP. Depletion of endopeptidase FURIN increased the plasma membrane-associated GFP intensity but not the Golgi-associated GFP intensity. (**H**) Representative images of plasma membrane- and lysosome-associated SLC23A1-GFP. Whereas depletion of GalNAc transferase GALNT2 increased GFP signals overall, depletion of SYVN1 led to a pronounced increase of GFP overlapping with the lysosomal RFP reference (see Fig. 6D for quantifications). (**I**) Representative images of plasma membrane- and vesicle-associated SLC31A1-GFP. Depletion of syntaxin-7 increased overall GFP signal while depletion of WDR6 increased GFP intensity at the plasma membrane (see Fig. 6D for quantifications).

 © The Author(s)
